# Open-source hardware platform for automated, non-invasive plant phenotyping through combined RGB and thermal imaging

**DOI:** 10.1016/j.ohx.2026.e00846

**Published:** 2026-10-05

**Authors:** Marcel Bentancor, Esteban Casaretto, Omar Borsani, Gastón Quero, Mercedes Muñoz

**Affiliations:** a Laboratorio de Biología Molecular Vegetal, Instituto de Química Biológica, Facultad de Ciencias, Universidad de la República. Iguá 4225, 11400 Montevideo, Uruguay; b Departamento de Biología Vegetal, Facultad de Agronomía, Universidad de la República. Av. Garzón 809, 12900 Montevideo, Uruguay; c Instituto de Computación, Facultad de Ingeniería, Universidad de la República. Av. Julio Herrera y Reissig 565, 11300 Montevideo, Uruguay

**Keywords:** Plant phenotyping, Thermal imaging, Visible-light imaging, Automated phenotyping platform

## Abstract

We present an open-source, modular plant phenotyping platform for non-invasive monitoring of small to medium-sized plants in growth chambers. The system integrates an RGB camera and a FLIR Lepton 3.5 thermal module on an automated two-axis Cartesian gantry capturing overhead images at 24 predefined positions. A key design feature is the use of Hall effect sensors with permanent magnets for discrete position referencing, providing robust, recalibration-free positioning without complex mechanical systems such as lead screws, belt-driven encoders, or step-counting alone. The platform uses 3D-printed components, a Raspberry Pi, stepper motors with rack-and-pinion transmission, and commonly available hardware store materials. Automated image acquisition scheduling increases temporal resolution and eliminates errors from prolonged manual measurements. Companion software enables homography-based projection of RGB segmentation masks onto thermal images and interactive exploration and quantitative analysis of thermographic data. As proof of concept, a multi-day water deficit experiment with soybean plants was conducted. RGB images were segmented using a U-Net deep learning model, and projected leaf area dynamics clearly discriminated between irrigated and drought-stressed plants. Thermal imaging revealed canopy temperature differences consistent with stomatal closure under water deficit.

## Graphical abstract

**Figure:**
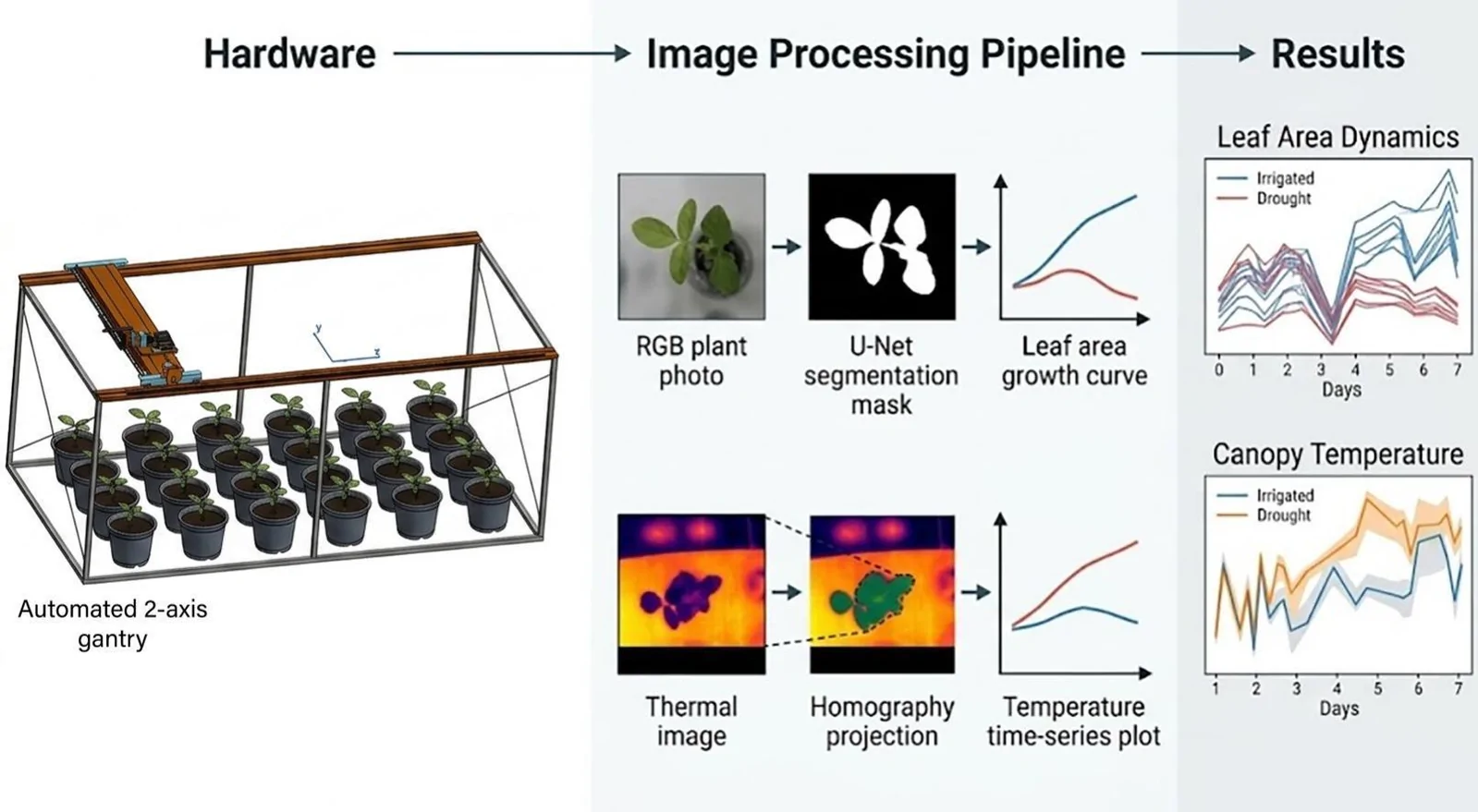

Specifications table

**Table:** 

Hardware name	Open-source plant phenotyping platform with RGB and thermal imaging
Subject area	● Environmental, planetary and agricultural sciences
Hardware type	● Imaging tools
Closest commercial analog	No direct commercial analog is available. While high-end systems such as those from PSI (e.g., PlantScreen Robotic XYZ) and Phenospex (e.g., TraitFinder) provide automated multimodal imaging in controlled environments, no low-cost (<USD 1,500), open-source, modular gantry system is currently available for routine RGB and thermal phenotyping in standard growth chambers.
Open source license	Creative Commons Attribution-ShareAlike 4.0 International License
Cost of hardware	USD 1110
Source file repository	https://doi.org/10.17632/n4cbs5kd8v

## 1 Hardware in context

The post-genomic era has generated an unprecedented availability of genomic and transcriptomic data in plant biology; however, this growth has not been matched by equivalent advances in the phenotypic characterization of genes and their regulatory pathways [1], [2], [3]. The complex interaction between genotype and environment that defines the phenotype demands tools capable of capturing dynamic and multiscalar processes. In this context, the use of predictive models [4], the incorporation of automated data acquisition technologies and the use of artificial intelligence algorithms have become central elements in accelerating the functional understanding of plant traits [5], [6], [7]. Nevertheless, these approaches require large volumes of high-quality phenotypic data, which has driven the development of automated, high-frequency measurement systems [8].

In functional genomics studies, especially when working with genetically modified plants, the initial evaluation stages are usually carried out in growth chambers. These environments allow for intensive screening and the selection of only the most promising genotypes before advancing to greenhouse or field trials, which demand greater investments of time, resources, and regulatory approvals [9], [10]. To address this need, various automated phenotyping platforms have been developed, incorporating multispectral cameras, environmental sensors, and robotic systems for the non-destructive capture of physiological and morphological traits [11].

Despite their capabilities, commercial platforms often present high costs and rely on specialized suppliers, which limits their accessibility, particularly in countries with less technological infrastructure. Furthermore, their level of customization is generally low, restricting their adaptation to specific experimental protocols [11]. In contrast, the recent development of open scientific hardware, together with the availability of programmable boards and digital manufacturing techniques, has favored the creation of more flexible, collaborative, and lower-cost solutions [12], [13], [14]. These advances have enabled the construction of highly customizable phenotyping platforms, capable of integrating into diverse experimental workflows [15], [16], [17].

Current plant phenotyping platforms span a wide range of operational scales, from modular multispectral systems to high-throughput 3D scanners. Commercial examples include PlantScreen™ platforms (PSI), which integrate RGB, chlorophyll fluorescence, hyperspectral, and thermal sensors, with costs that can exceed USD 100,000 depending on configuration [11]; the PlantEye F500 system (Phenospex), a rail-mounted 3D laser scanner for rapid architectural measurements exceeding 1,000 plants per hour, with approximate costs of USD 147,000 [19]; and the LemnaTec Scanalyzer family, which incorporates automated pot transport, weighing stations, and multispectral modules, with total costs that may approach one million USD depending on configuration. At the other end of the spectrum, open-hardware solutions such as the prototypes described by Bagley et al. [20], based on NEMA-17 motors and Arduino controllers, enable basic automated acquisition in growth chambers at substantially lower cost, although without integrated multimodal imaging. Likewise, the MVS-Pheno v1 system provides a portable intermediate alternative for multi-view reconstruction at approximately USD 7,600 [21].

Within this framework, the platform developed in the present study is positioned not as a replacement for high-throughput commercial infrastructures, but as a complementary solution for automated phenotyping in growth chambers and potentially in small-scale greenhouse setups. It combines a sufficient degree of automation to generate high-frequency phenotypic data with a modular, low-cost design that is accessible, reproducible, and adaptable to laboratories with limited resources.

A key dimension of these platforms is the integration of optical sensors. Infrared thermography (IRT) has established itself as a fundamental tool for assessing the physiological status of plants, by allowing indirect estimation of stomatal conductance and transpiration through the measurement of leaf temperature [22]. Under water or heat stress, stomatal closure reduces heat loss through evaporation, raising leaf temperature; this phenomenon has been extensively documented in field and laboratory studies since the early 2000s, including applications in irrigation scheduling [23]. More recently, Pineda et al. [24] reviewed the use of IRT cameras for the detection of biotic and abiotic stress, highlighting their utility when adequate environmental corrections are integrated.

In parallel, RGB images, combined with photogrammetric analysis and extraction of indices derived from the visible spectrum, allow the quantification of leaf area, architecture, projected biomass, pigmentary changes, and senescence. Among these indices, recently the Green Index (GI) has been proposed as an improved RGB-based metric for quantifying leaf greenness, showing strong correlations with chlorophyll content across a variety of experimental contexts [25]. The simultaneous integration of IRT and RGB offers a multimodal characterization of plant stress, by combining physiological information related to water status with morphological and pigmentary traits. This complementarity has proven to be particularly effective in phenotyping studies under both controlled and field conditions; for example, Stutsel et al. [26] demonstrated that the combination of thermal and RGB sensors mounted on UAVs enables the early detection of salt stress impact before the appearance of visible symptoms. Overall, the integration of infrared thermography and RGB imaging represents a robust, non-invasive, and scalable approach for the detection and analysis of plant stress, constituting an essential component of modern high-throughput phenotyping platforms.

More broadly, existing phenotyping platforms address distinct experimental aims rather than a single universal use case. Some open-source systems prioritize irrigation control or soil water monitoring [18], [27], whereas others, such as the SPOT facility, emphasize extensive multisensor integration including hyperspectral, thermal, and LiDAR imaging [28]. At a larger scale, highly robotized facilities are designed for the translation of controlled-environment traits to field performance through environmental modelling and genomic prediction [29]. Additional low-cost systems rely on simple robotic vectors carrying specific sensors for targeted tasks along a single axis [20]. This diversity highlights that platform design should be evaluated in relation to the experimental niche it is intended to serve.

While these systems demonstrate the increasing sophistication of automated phenotyping technologies, many rely on complex infrastructures, proprietary subsystems, or tightly integrated environmental control, which may reduce their accessibility and limit their flexibility for routine experiments in growth chambers.

The platform presented here addresses a complementary niche by prioritizing simplicity, modularity, and reproducibility for controlled-environment phenotyping. It consists of a low-cost two-axis Cartesian system that combines RGB and thermal imaging for non-invasive, high-frequency screening of small- to medium-sized plants under laboratory conditions. Unlike positioning systems that rely on belt-driven encoders, lead screws, or cumulative step counting, the platform uses Hall effect sensors and permanent magnets as discrete positional references, enabling robust positioning while tolerating occasional step losses without recalibration. Movement across the experimental area is achieved through a rack-and-pinion transmission driven by stepper motors, allowing repeated scanning of a predefined plant grid. Companion software tools support homography-based registration between RGB and thermal images and the quantitative analysis of both modalities. By separating image acquisition from environmental control, the platform maintains a minimal and accessible architecture that facilitates replication, sensor substitution, and adaptation to diverse experimental workflows, while supporting dense temporal sampling.

As summarized in Table 1, the present platform is, to our knowledge, the only open-source system that combines two-axis automated positioning, integrated RGB and thermal imaging, and cross-modal registration software at a cost below USD 1200.

**Table 1:** Comparison with previously published plant phenotyping platforms.

Platform / year	Cost (USD)	Movement Axes	Thermal sensing	Positioning Method	Target Environment
RasPiPheno / 2024 [30]	<200 (per setup)	Fixed (top and side modules)	None	Fixed positions (PhenoRig / PhenoCage)	Growth chamber / Greenhouse
Kim et al. / 2023 [27]	874 (sensing system only)	None (fixed)	IR thermometer (point)	N/A	Growth chamber
Bethge et al./ 2023 [15]	∼3,200	3 (x, y, z)	Thermal camera (limited validation)	Stepper motors + belt drive	*In vitro* culture vessels
SPOT / 2023 [28]	Up to 38,000	2 (x, y)	Thermal camera	Stepper motors + lead screws	Laboratory / Controlled environment
Takara et al./ 2021 [18]	∼1,276	1 (x)	None	Belt drive + Hall effect + magnets	Growth chamber
Bagley et al. / 2020 [20]	$300–$900 (excluding cameras and PC)	1 (x)	Thermal camera	Stepper motors + belt drive	Growth chamber
PhenoBox / 2018 [16]	3300	Turntable (rotation)	None	Turntable + fixed camera	Controlled environment
Phenotiki / 2017 [17]	∼220	None (fixed)	None	Fixed overhead camera	Controlled environment (rosettes)
Present platform	1110	2 (x, y)	Thermal camera	Rack and pinion + Hall effect + magnets	Growth chamber

Cost estimates for previously published platforms exclude cameras and computing equipment unless otherwise noted by the original authors. The cost reported for the present platform (USD 1110) includes all components listed in the Bill of Materials, including the Raspberry Pi, RGB camera, lens, and thermal imaging module; only a standard monitor, keyboard, and mouse for initial setup, as well as soldering supplies, tools, and labor, are excluded. “None” indicates the absence of a given feature; “N/A” indicates that the category is not applicable to the platform’s design.

Cost estimates for previously published platforms exclude cameras and computing equipment unless otherwise noted by the original authors. The cost reported for the present platform (USD 1110) includes all components listed in the Bill of Materials, including the Raspberry Pi, RGB camera, lens, and thermal imaging module; only a standard monitor, keyboard, and mouse for initial setup, as well as soldering supplies, tools, and labor, are excluded. “None” indicates the absence of a given feature; “N/A” indicates that the category is not applicable to the platform’s design.

## 2 Hardware description

The present hardware was conceived as a modular, non-invasive, and open-design plant phenotyping platform specifically oriented toward controlled cultivation environments such as growth chambers (Fig. 1). Rather than reproducing the scale or infrastructure of commercial automated phenotyping facilities, the system was designed to provide a customizable, easily replicable, and low-cost alternative for routine controlled-environment experiments in research groups with limited resources.

**Fig. 1:**
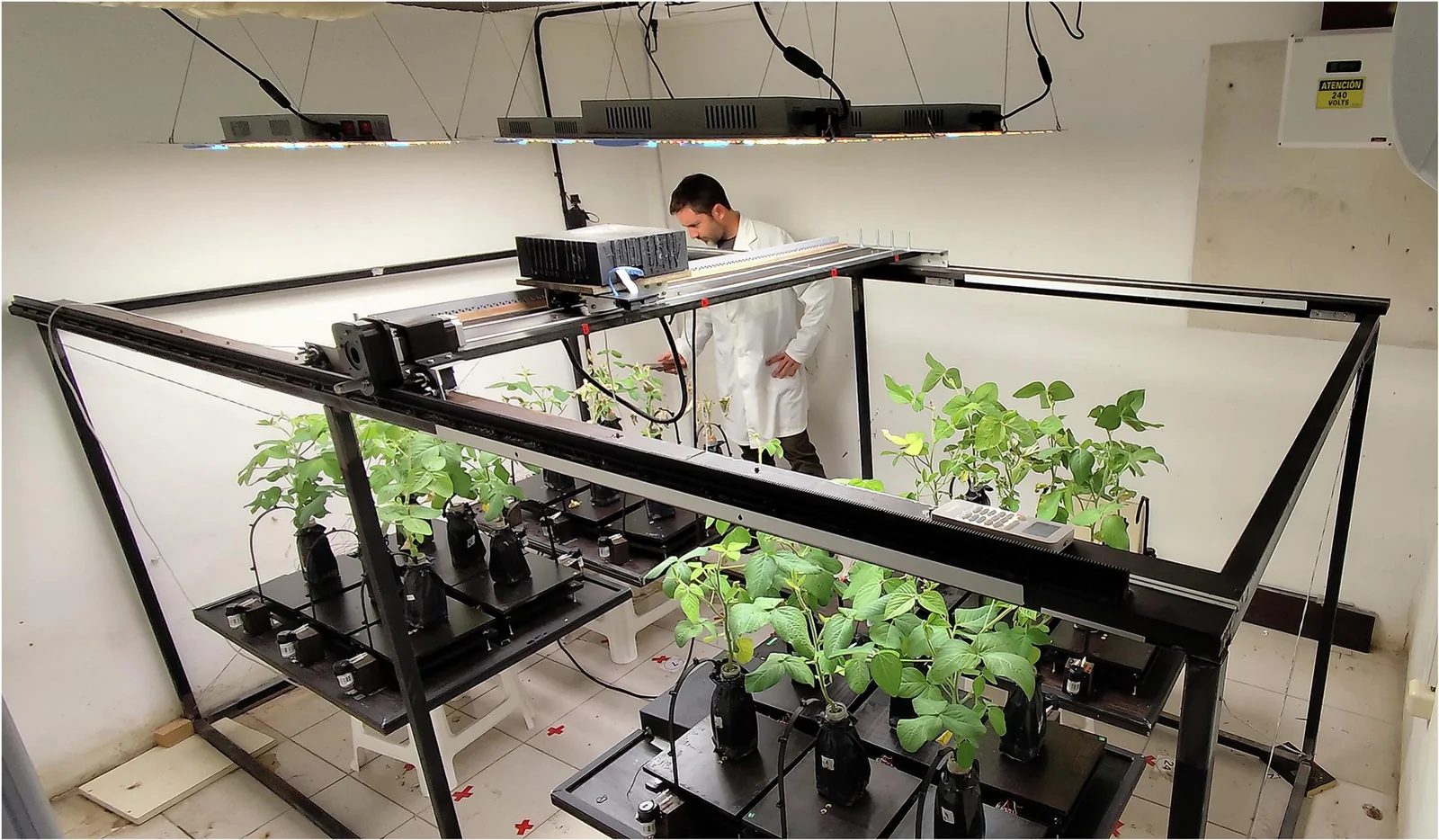
General view of the plant phenotyping platform installed in a plant growth chamber with soybean plants (*Glycine max*).

Automated phenotyping platforms can be broadly classified into two architectural paradigms: plant-to-sensor systems, in which plants are transported to a fixed imaging station via conveyors or robotic handlers, and sensor-to-plant systems, in which the imaging unit moves over stationary plants. Plant-to-sensor architectures enable standardized imaging conditions and high throughput, but typically require complex mechanical infrastructure for pot handling, transport, and repositioning, leading to increased cost, mechanical wear, and long-term maintenance requirements.

The platform presented here adopts a sensor-to-plant approach, incorporating an automated overhead gantry that allows the camera to travel over stationary plants. This design enables the acquisition of high-frequency time series while reducing manual intervention. In contrast to commercial systems that rely on conveyors, robotic arms, belts, or rotating platforms, the proposed structure and motion transmission mechanisms were intentionally designed to be robust, simplified, and suitable for assembly by non-specialized personnel. In this regard, a rack-and-pinion motion transmission mechanism was chosen, driven by stepper motors and equipped with Hall effect sensors to maintain movement precision, without the need for recalibration of the motion system, while also being robust against occasional loss of steps or traction.

Two types of cameras are included: an RGB camera for visible imaging and a thermal camera for capturing surface temperature data or thermal patterns of the plants.

The platform is controlled by a Raspberry Pi board, connected to stepper motors, position sensors, and communication with a central computer or local network. Thanks to the 3D printing of supports and parts, as well as the use of common hardware store components, users can build and modify the platform without depending on exclusive manufacturers. Through the automation of displacement, capture, and image storage, the data acquisition frequency is increased. For example, every hour, every 30 min, or even more frequently, and the human errors or biases that typically arise during prolonged manual measurements are minimized.

The modular design allows for interchanging sensors, adjusting camera height, or modifying the movement trajectory, which enables adapting the system to different plant sizes and pot formats. This flexibility contrasts with many industrial platforms, which are usually optimized for specific experimental configurations and offer a limited degree of customization.

This equipment consists of a rectangular parallelepiped-shaped structure made of square iron pipes (Fig. 2). This structure supports an automated displacement mechanism in a two-axis Cartesian system (x, y). A head travels over this structure in both directions. This head stops at 24 predefined positions where the plants to be studied are placed. Once the head is positioned there, images are captured in the visible spectrum (RGB) and thermographic images are taken using a Lepton 3.5 module (FLIR). The stopping positions are defined by the placement of magnets in both directions (Fig. 3), which are detected by Hall effect sensors. Both motion control and image acquisition are managed by a Raspberry Pi 3B + board. This board also enables remote monitoring and control of the platform via a WiFi network connection.

**Fig. 2:**
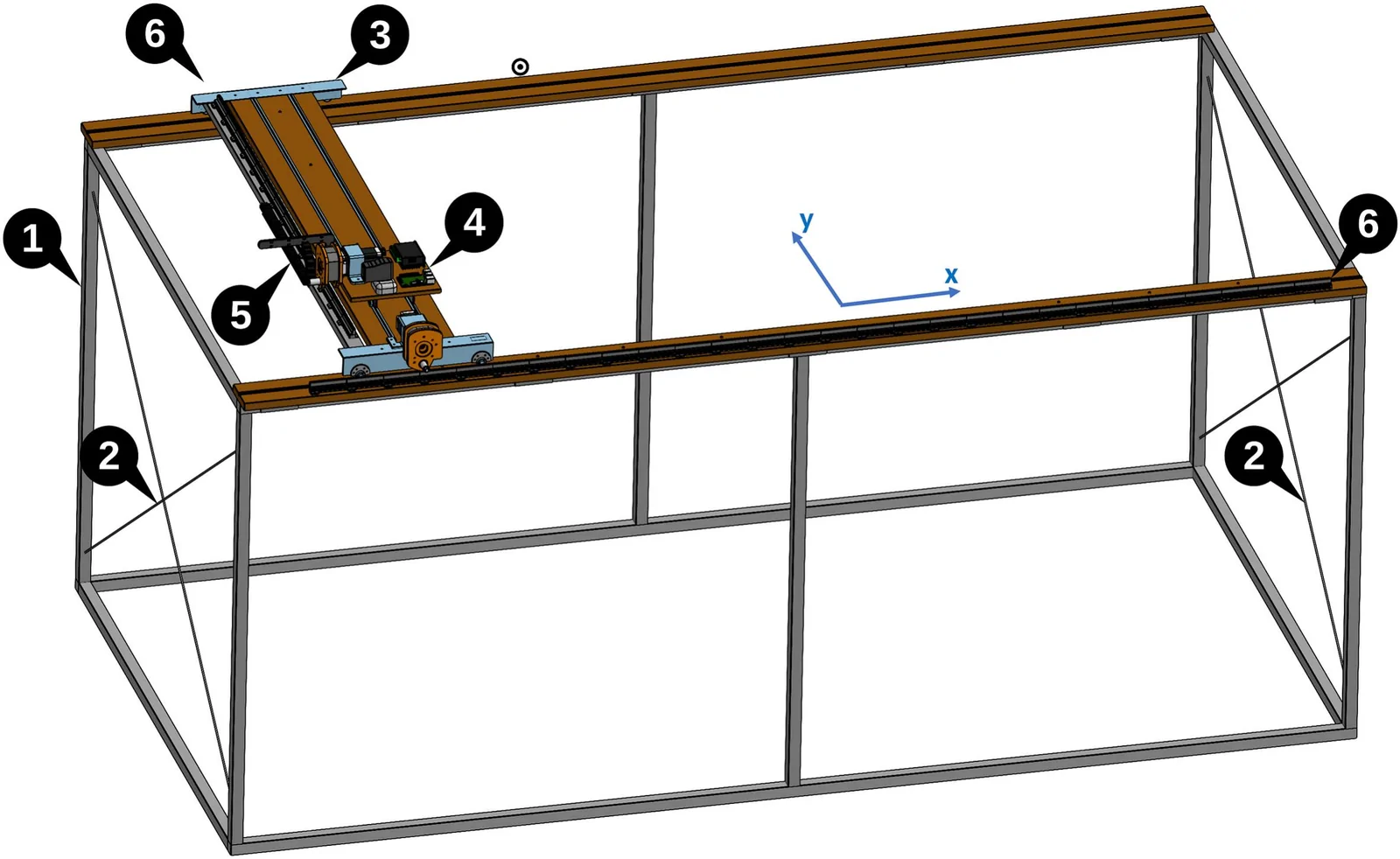
Structure of the plant phenotyping platform. 1, main metal frame. 2, tensioners. 3, macro-carriage. 4, micro-carriage. 5, cable carrier chain. 6, racks for traction of both carriages. The orthogonal x and y displacement axes of the macro- and micro-carriage are indicated.

**Fig. 3:**
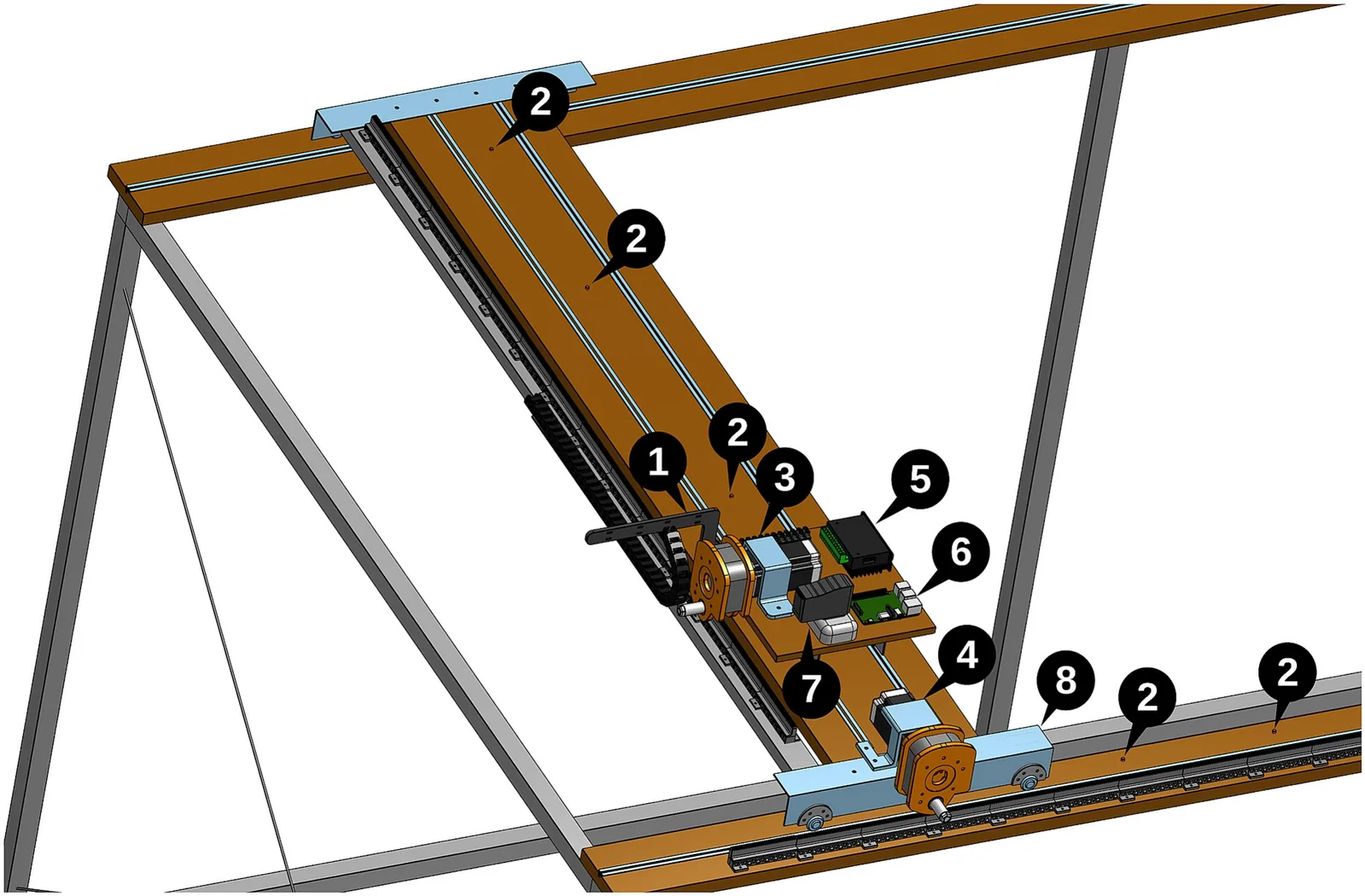
Layout of the main elements of the platform. 1, support for guiding the cables coming from the micro-carriage. 2, magnets indicating the stopping positions on the x and y axes (one of the magnets in this view is hidden by the micro-carriage). 3, holder and motor for movement along the y axis. 4, holder and motor for movement along the x axis. In this section, on the underside of the macro-carriage, is the driver for the x-axis movement motor. 5, driver for the y-axis movement motor. 6, Raspberry Pi board. 7, electrical outlet and adapter for Raspberry Pi power supply. 8, macro-carriage.

For researchers, some of the ways in which this equipment can assist them are:

• It enables high-frequency monitoring of plant growth and physiological status in growth chambers, which facilitates the detection of early changes resulting from experimental treatments.
• It facilitates the screening of genetically modified lines or gene function assays under controlled conditions before scaling to the field, reducing time and resources for field trials.
• It offers modular and customizable hardware: researchers can adapt sensors, movement trajectories, or system size according to their model plant, budget, or experimental needs.
• It reduces dependence on costly commercial platforms, promoting its use in laboratories with limited resources (such as in non-central countries) and fostering open standards.
• It promotes reproducibility by automating data capture and storage, minimizing human variability in measurements and enabling denser time series.

## 3 Design files summary

The design files associated with the platform are listed in Table 2.

**Table 2:** Summary of design files, file types, licenses and repository locations.

**Design file name**	**File type**	**Open source license**	**Location of the file**
file1a_cover_motor_bridge.stl	STL file	Creative Commons Attribution-ShareAlike 4.0 International License	https://doi.org/10.17632/n4cbs5kd8v
file1b_cover_motor_front.stl	STL file	Creative Commons Attribution-ShareAlike 4.0 International License	https://doi.org/10.17632/n4cbs5kd8v
file1c_cover_motor_back.stl	STL file	Creative Commons Attribution-ShareAlike 4.0 International License	https://doi.org/10.17632/n4cbs5kd8v
file2_pulley_8m_nema23.stl	STL file	Creative Commons Attribution-ShareAlike 4.0 International License	https://doi.org/10.17632/n4cbs5kd8v
file3_pinion.stl	STL file	Creative Commons Attribution-ShareAlike 4.0 International License	https://doi.org/10.17632/n4cbs5kd8v
file4_long_rack.stl	STL file	Creative Commons Attribution-ShareAlike 4.0 International License	https://doi.org/10.17632/n4cbs5kd8v
file5_long_rack_guide.stl	STL file	Creative Commons Attribution-ShareAlike 4.0 International License	https://doi.org/10.17632/n4cbs5kd8v
file6_thermal_cover_back.stl	STL file	Creative Commons Attribution-ShareAlike 4.0 International License	https://doi.org/10.17632/n4cbs5kd8v
file6_thermal_cover_front.stl	STL file	Creative Commons Attribution-ShareAlike 4.0 International License	https://doi.org/10.17632/n4cbs5kd8v
file7_cameras_support.stl	STL file	Creative Commons Attribution-ShareAlike 4.0 International License	https://doi.org/10.17632/n4cbs5kd8v
file8_cylindrical_adapter.stl	STL file	Creative Commons Attribution-ShareAlike 4.0 International License	https://doi.org/10.17632/n4cbs5kd8v
file9_Hall_sensor_support.stl	STL file	Creative Commons Attribution-ShareAlike 4.0 International License	https://doi.org/10.17632/n4cbs5kd8v
file10a_main_cover.stl	STL file	Creative Commons Attribution-ShareAlike 4.0 International License	https://doi.org/10.17632/n4cbs5kd8v
file10b_main_cover.stl	STL file	Creative Commons Attribution-ShareAlike 4.0 International License	https://doi.org/10.17632/n4cbs5kd8v
file11_support_motor_y.stl	STL file	Creative Commons Attribution-ShareAlike 4.0 International License	https://doi.org/10.17632/n4cbs5kd8v
file12_support_motor_x.stl	STL file	Creative Commons Attribution-ShareAlike 4.0 International License	https://doi.org/10.17632/n4cbs5kd8v
file13_cable_support.stl	STL file	Creative Commons Attribution-ShareAlike 4.0 International License	https://doi.org/10.17632/n4cbs5kd8v
dual_capture.py	Python script	Creative Commons Attribution-ShareAlike 4.0 International License	https://doi.org/10.17632/n4cbs5kd8v
movement_functions.py	Python script	Creative Commons Attribution-ShareAlike 4.0 International License	https://doi.org/10.17632/n4cbs5kd8v
main_gui.py	Python script	Creative Commons Attribution-ShareAlike 4.0 International License	https://doi.org/10.17632/n4cbs5kd8v
motors.py	Python script	Creative Commons Attribution-ShareAlike 4.0 International License	https://doi.org/10.17632/n4cbs5kd8v
reset_lepton.py	Python script	Creative Commons Attribution-ShareAlike 4.0 International License	https://doi.org/10.17632/n4cbs5kd8v
1_apply_geometric_correction.py	Python script	Creative Commons Attribution-ShareAlike 4.0 International License	https://doi.org/10.17632/n4cbs5kd8v
2_plant_segmentation_training.ipynb	Jupyter notebook	Creative Commons Attribution-ShareAlike 4.0 International License	https://doi.org/10.17632/n4cbs5kd8v
3_mask_postprocessing_adaptive_bbox.ipynb	Jupyter notebook	Creative Commons Attribution-ShareAlike 4.0 International License	https://doi.org/10.17632/n4cbs5kd8v
4_leaf_area_temporal_analysis.py	Python script	Creative Commons Attribution-ShareAlike 4.0 International License	https://doi.org/10.17632/n4cbs5kd8v
5_image_mask_viewer_napari.py	Python script	Creative Commons Attribution-ShareAlike 4.0 International License	https://doi.org/10.17632/n4cbs5kd8v
1_homography_calibrator.py	Python script	Creative Commons Attribution-ShareAlike 4.0 International License	https://doi.org/10.17632/n4cbs5kd8v
2_palette_navigator.py	Python script	Creative Commons Attribution-ShareAlike 4.0 International License	https://doi.org/10.17632/n4cbs5kd8v
3_generate_statistics.py	Python script	Creative Commons Attribution-ShareAlike 4.0 International License	https://doi.org/10.17632/n4cbs5kd8v
4_plot_temperatures.py	Python script	Creative Commons Attribution-ShareAlike 4.0 International License	https://doi.org/10.17632/n4cbs5kd8v
homography_rgb_to_thermal.json	JSON file	Creative Commons Attribution-ShareAlike 4.0 International License	https://doi.org/10.17632/n4cbs5kd8v
plant_segmentation_model.pth	PyTorch model file	Creative Commons Attribution-ShareAlike 4.0 International License	https://doi.org/10.17632/n4cbs5kd8v
calibration_640x480.npz	NumPy archive	Creative Commons Attribution-ShareAlike 4.0 International License	https://doi.org/10.17632/n4cbs5kd8v
README.md	Documentation file	Creative Commons Attribution-ShareAlike 4.0 International License	https://doi.org/10.17632/n4cbs5kd8v
README_image_processing.md	Documentation file	Creative Commons Attribution-ShareAlike 4.0 International License	https://doi.org/10.17632/n4cbs5kd8v
README_platform_operation.md	Documentation file	Creative Commons Attribution-ShareAlike 4.0 International License	https://doi.org/10.17632/n4cbs5kd8v
requirements.txt (platform_operation)	Text file	Creative Commons Attribution-ShareAlike 4.0 International License	https://doi.org/10.17632/n4cbs5kd8v
requirements.txt (image_processing)	Text file	Creative Commons Attribution-ShareAlike 4.0 International License	https://doi.org/10.17632/n4cbs5kd8v
calibrate_pattern_images.py	Python script	Creative Commons Attribution-ShareAlike 4.0 International License	https://doi.org/10.17632/n4cbs5kd8v
geometric_calibration.py	Python script	Creative Commons Attribution-ShareAlike 4.0 International License	https://doi.org/10.17632/n4cbs5kd8v
dual_capture_compatible.py	Python script	Creative Commons Attribution-ShareAlike 4.0 International License	https://doi.org/10.17632/n4cbs5kd8v
pattern_images.zip	ZIP file	Creative Commons Attribution-ShareAlike 4.0 International License	https://doi.org/10.17632/n4cbs5kd8v
experiment.zip	ZIP file	Creative Commons Attribution-ShareAlike 4.0 International License	https://doi.org/10.17632/n4cbs5kd8v
platform_operation_demo.mp4	MP4 file	Creative Commons Attribution-ShareAlike 4.0 International License	https://doi.org/10.17632/n4cbs5kd8v

3D printing models:

file1a_cover_motor_bridge.stl, intermediate piece of the cover for the motion transmission mechanism.

file1b_cover_motor_front.stl, front piece of the cover for the motion transmission mechanism.

file1c_cover_motor_back.stl, rear piece of the cover for the motion transmission mechanism.

file2_pulley_8m_nema23.stl, pulley.

file3_pinion.stl, pinion.

file4_long_rack.stl, structural element of the rack for macro- and micro-carriage traction.

file5_long_rack_guide.stl, 3D model of the guide piece for correctly aligning the racks during their installation on the platform.

file6_thermal_cover_back.stl, rear part of the cover for the thermographic module.

file6_thermal_cover_front.stl, front part of the cover for the thermographic module.

file7_cameras_support.stl, support for the RGB camera and thermographic module.

file8_cylindrical_adapter.stl, cylindrical adapter for adjusting the shaft of the motion transmission mechanism.

file9_Hall_sensor_support.stl, support for mounting the Hall effect sensor.

file10a_main_cover.stl and file10b_main_cover.stl, micro-carriage cover and its support, respectively.

file11_support_motor_y.stl and file12_support_motor_x.stl, holder pieces for the y- and x-axis motors, respectively.

file13_cable_support.stl, cable guide, which is mounted on the micro-carriage.

The software accompanying the platform is organized into two independent modules, each designed to run on a different computing environment. The first module runs directly on the Raspberry Pi 3B + board mounted on the platform and handles real-time operations: motor control, Hall effect sensor reading, scheduling of acquisition routines, and synchronized capture of RGB and thermal images. This module is composed of five Python scripts (main_gui.py, movement_functions.py, dual_capture.py, motors.py, and reset_lepton.py) and operates autonomously once configured, requiring no external computer during data acquisition. The second module runs offline on a standard desktop or laptop computer and performs all post-acquisition image processing, including geometric correction, deep learning-based segmentation, mask post-processing, homography calibration, thermal mask curation, and statistical analysis. This module comprises nine Python scripts and two Jupyter notebooks, with separate dependency files for each environment. The two modules are connected through the image files stored on the Raspberry Pi’s filesystem, which can be transferred to the processing computer via the local network, USB storage, or any standard file transfer method. Each module includes its own README documentation file and requirements.txt specifying the necessary dependencies.

Python scripts for operating the platform:

README.md.

Top-level documentation file providing a general overview of the repository structure, a summary of the 3D-printed mechanical parts and both software modules (platform operation and image processing), the system specifications, the experimental treatment layout, dependency installation instructions, and links to the detailed documentation of each module.

Requirements.txt (platform_operation)

Plain-text list of the three Python package dependencies (RPi.GPIO, numpy, opencv-python) with minimum version constraints for the platform operation scripts, installable via pip.

README_platform_operation.md

Documentation file describing the hardware requirements, GPIO pin assignments, software dependencies, repository structure, installation instructions, usage, output directory layout, traversal pattern, and calibration notes for the platform operation scripts.

main_gui.py

Tkinter-based graphical interface that allows the user to program one or more daily execution times (HH:MM) and to manually position both carriages using joystick-style directional buttons, with selectable continuous or magnet-to-magnet movement modes.

movement_functions.py

Implements the full autonomous phenotyping cycle: S-pattern traversal of the 6 × 4 measurement grid, dual-image capture at each of the 24 positions, and a Hall-sensor-based homing routine that returns the macro-carriage to the rest position at the end of each session.

dual_capture.py

Acquires a synchronized RGB image (via libcamera-still) and a FLIR Lepton thermal frame (via ffmpeg/V4L2) at a given (X, Y) grid position, saving outputs in multiple formats including RAW Y16, NPY, 16-bit TIFF, JPEG, and a per-pixel temperature CSV.

motors.py

Low-level motor control library that provides thread-safe primitives for both continuous stepping and magnet-to-magnet jumps on the x and y axes, along with a mock GPIO fallback that enables off-board testing without a Raspberry Pi.

reset_lepton.py

Standalone utility that recovers a non-responsive FLIR Lepton camera without a system reboot by terminating active ffmpeg processes and cycling the uvcvideo kernel module.

Python scripts for RGB image processing:

README_image_processing.md

Documentation file describing the image processing pipeline, script dependencies, execution order, directory structure conventions, and usage instructions for the RGB and thermal image processing scripts.

requirements.txt (image_processing)

Plain-text list of the Python package dependencies for the image processing scripts (numpy, opencv-python, pandas, matplotlib, Pillow, napari, scikit-image, tqdm), with minimum version constraints, installable via pip.

1_apply_geometric_correction.py

Applies lens distortion correction to RGB images using intrinsic camera parameters stored in a NumPy calibration file (.npz). Processes all images found in a user-selected folder, generating undistorted outputs with cropped valid regions. Includes a Tkinter-based configuration dialog for selecting input folder, calibration file, and output location.

2_plant_segmentation_training.ipynb

Jupyter notebook implementing the training pipeline for the U-Net segmentation model (ResNet34 encoder). Covers data loading from Supervisely-exported annotations, dataset splitting (80/20 train/validation), model training with loss and IoU/Dice metric tracking, and inference on the full image set to generate binary segmentation masks.

3_mask_postprocessing_adaptive_bbox.ipynb

Jupyter notebook that post-processes the raw U-Net segmentation masks to isolate the central plant in each image. Applies adaptive bounding box filtering and morphological watershed separation to remove neighboring plants from the field of view.

4_leaf_area_temporal_analysis.py

Calculates projected leaf area (in pixels) from the post-processed RGB segmentation masks for each plant across all timepoints. Generates a CSV with per-plant area time series and a publication-quality plot distinguishing irrigated (R) and drought-stressed (S) treatments.

5_image_mask_viewer_napari.py

Napari-based viewer for side-by-side inspection of RGB images and their corresponding segmentation masks. Provides interactive paint and eraser tools for manual correction of masks that could not be fully resolved by automated post-processing, with in-place saving of edited masks.

plant_segmentation_model.pth

Pre-trained weights of the U-Net segmentation model (ResNet34 encoder, 50 epochs on 100 manually annotated images) saved as a PyTorch state dictionary. Loaded by 2_plant_segmentation_training.ipynb during inference to apply segmentation to the experimental dataset without retraining.

calibration_640x480.npz

NumPy archive containing the intrinsic camera matrix and lens distortion coefficients of the Raspberry Pi HQ Camera at 640 × 480 px (RMS reprojection error: 0.11 px). Loaded by 1_apply_geometric_correction.py to remove barrel distortion from RGB captures.

Python scripts for thermal image processing:

1_homography_calibrator.py

Graphical tool for computing the projective transformation (homography) between the geometrically corrected RGB images (597 × 431 px) and the FLIR Lepton thermal images (160 × 120 px). The user defines corresponding reference points on both image modalities, and the tool calculates, saves, and loads the homography matrix used to project RGB-derived segmentation masks onto the thermal image space.

2_palette_navigator.py

Interactive graphical interface for exploring thermographic images, visualizing homography-projected RGB masks overlaid on thermal data, manually editing mask boundaries to correct misalignments, adjusting color palettes, and inspecting per-pixel temperature distributions through histograms. Generates curated thermal masks used for subsequent temperature statistics extraction.

3_generate_statistics.py

Processes all thermographic images from the experiment and generates a CSV file with temperature statistics (min, max, mean, median, standard deviation, percentiles, IQR, coefficient of variation) for each plant and acquisition timepoint, using the curated thermal masks generated by 2_palette_navigator.py.

4_plot_temperatures.py

Generates publication-quality plots of canopy temperature dynamics, comparing irrigated and drought-stressed treatments over the experimental period using the statistics CSV produced by 3_generate_statistics.py.

homography_rgb_to_thermal.json

JSON file containing the pre-computed homography matrix, source and target image dimensions, and the corresponding reference point coordinates used to project RGB masks onto the thermal image space.

Python scripts and assets for camera geometric calibration:

calibrate_pattern_images.py

Entry-point script for the calibration. Loops over each resolution subfolder in pattern_images/, runs GeometricCalibrator on it, and writes the calibration in.npz,.json and.yaml plus two demo undistortion images.

geometric_calibration.py

Calibration library. Detects chessboard corners with subpixel refinement, calls cv2.calibrateCamera to obtain the intrinsic matrix and distortion coefficients, and reports the reprojection error per image and overall.

dual_capture_compatible.py

Raspberry Pi script that captures chessboard images at 640 × 480 and 1920 × 1080 px simultaneously via libcamera-still. Provides an interactive shell (preview, photo, quit); each photo writes one paired capture per resolution.

pattern_images.zip

64 chessboard images from the calibration session of this study: 32 at 640 × 480 px and 32 at 1920 × 1080 px. Unzip in place to obtain the pattern_images/ folder structure expected by calibrate_pattern_images.py. The target was the standard 9 × 6 internal-corner OpenCV chessboard (https://github.com/opencv/opencv/blob/4.x/doc/pattern.png).

Validation experiment dataset:

experiment.zip

Raw RGB and thermal captures from the validation experiment (Section 7), organized as experiment/YYYY-MM-DD_HHh/{picam,lepton}/, with one subfolder per acquisition session. Each thermal capture is stored in six complementary formats: .NPY.npy, .TIFF, .Y16, _color.jpg, _grayscale.jpg, and _temperature.csv. Unzip in place inside data/ to obtain the folder structure expected by the image-processing pipeline.

platform_operation_demo.mp4

The video shows the operation of the platform and follows the sequence of steps described in the operation instructions section. It contains no narration or soundtrack; all explanations are provided as on-screen text.

## 4 Bill of materials summary

The components required to build the platform are listed in Table 3.

**Table 3:** Bill of materials.

**Designator**	**Component**	**Number**	**Cost per unit − USD**	**Total cost −** **USD**	**Source of materials**	**Material type**
Item 1	Square steel tube, 30 × 30 × 2 × 3000 mm	4	15.50	62	Local hardware store	Metal
Item 2	Square steel tube, 30 × 30 × 2 × 1250 mm	6	6.50	39	Local hardware store	Metal
Item 3	Square steel tube, 30 × 30 × 2 × 1500 mm	4	7.80	31.2	Local hardware store	Metal
Item 4	Mild galvanized steel wire, 1.6 mm in diameter, 4000 mm length	4	0.2	0.8	Local hardware store	Metal
Item 5	Board with a cross-sectional area of 110 × 20 mm and an overall length of 3000 mm	2	5.5	11	Local hardware store	Organic
Item 6	L-shaped aluminum angle (length = 100 mm; flange width = 30 mm)	20	0.85	17	Local hardware store	Metal
Item 7	Solid rivets, 20 mm length × 2.5 mm nominal diameter	40	0.05	2	Local hardware store	Metal
Item 8	Wood screws (4 mm in diameter and 15 mm in length)	50	0.020	1	Local hardware store	Metal
Item 9	Aluminum guide rail (Meaton AL-1535/CD50) Nominal width of 14 mm and an overall length of 3000 mm, incorporating a precision-machined groove measuring 3 mm in width and 5 mm in depth	2	18	36	Local hardware store	Metal
Item 10	Wood screws (2.5 mm in diameter and 10 mm in length)	80	0.025	2	Local hardware store	Metal
Item 11	Magnets 5x2.8 mm	11	0.09	0.99	link ASIN: B0936M3WPK	Metal
Item 12	Board with a cross-sectional area of 220 × 20 mm and an overall length of 1530 mm	1	5.6	5.6	Local hardware store	Organic
Item 13	Board with a cross-sectional area of 40 × 10 mm and an overall length of 1530 mm	1	2	2	Local hardware store	Organic
Item 14	L-shaped aluminum angle (length = 400 mm; flange width = 50 mm)	2	5	10	Local hardware store	Metal
Item 15	Screws (8 mm in diameter and 75 mm in length)	8	0.40	3.2	Local hardware store	Metal
Item16	Nuts for item 15	8	0.075	0.6	Local hardware store	Metal
Item 17	Washers for item 15	8	0.075	0.6	Local hardware store	Metal
Item 18	L-shaped aluminum angle (length = 1530 mm; flange width = 40 x 20 mm)	2	18.5	37	Local hardware store	Metal
Item 19	Sliding door roller set (Ducasse model AL-1535)	8	3.60	28.8	Local hardware store	Metal
Item 20	M5 x 30 mm Hexagon head bolt	32	0.10	3.2	Local hardware store	Metal
Item 21	Wing nuts for item 20	32	0.25	8	Local hardware store	Metal
Item 22	Aluminum guide rail (Meaton AL-1535/CD50) Nominal width of 14 mm and an overall length of 1540 mm, incorporating a precision-machined groove measuring 3 mm in width and 5 mm in depth	2	9	18	Local hardware store	Metal
Item 23	NEMA 23 stepper motor, 425 oz·in, 3 A, 112 mm	2	39.99	79.98	link ASIN: B00PNEPW4C	Other
Item 24	FMD2740C CNC 2 Phase Stepper Motor Driver 50VDC 4A 128 Microstep	2	37.07	74.14	link ASIN: B0FS1KGG1V	Other
Item 25	Hall Effect Sensor Magnetic Sensor Module	2	1.92	3.84	link ASIN: B07VDJSP3D	Other
Item 26	608RS deep groove ball bearing (8 × 22 × 7 mm)	4	0.65	2.6	link ASIN: B092TZY939	Metal
Item 27	GT2 closed-loop timing belt (170 mm length × 6 mm width)	2	1.0	2.0	link ASIN: B0CMHW5ZDF	Polymer
Item 28	M8 × 90 mm hexagon head bolt	2	1	2	Local hardware store	Metal
Item 29	Self-locking nut for item 28	2	0.11	0.22	Local hardware store	Metal
Item 30	Threaded rod, 4 mm diameter, 55 mm length	6	0.15	0.9	Local hardware store	Metal
Item 31	Nuts for Item 30	12	0.05	0.6	Local hardware store	Metal
Item 32	Square board 24 cm on each side and 1.5 cm thick	1	1	1	Local hardware store	Organic
Item 33	L-shaped aluminum angle (length = 80 mm; flange width = 45 mm)	4	1	4	Local hardware store	Metal
Item 34	Power outlet	1	3	3	Local hardware store	Polymer
Item 35	Raspberry Pi 3B^+^ (kit with electrical adapter included)	1	79.89	79.89	link ASIN: B08G8S63SL	Other
Item 36	36 V, 11A DC Power Supply	1	26.98	26.98	link ASIN: B01IOQ18G6	Other
Item 37	Plastic Cable Wire Carrier Drag Chain	1	9.99	9.99	link ASIN: B07QVKL9VL	Polymer
Item 38	L-shaped aluminum angle (length = 400 mm; flange width = 20 mm)	1	2	2	Local hardware store	Metal
Item 39	Raspberry Pi HQ Camera	1	75.59	75.59	link ASIN: B08LHJR3K4	Other
Item 40	M2,5 x 18 mm Hexagon head bolt	4	0.25	1	Local hardware store	Metal
Item 41	Hexagon nut for item 40	4	0.12	0.48	Local hardware store	Metal
Item 42	Arducam HQ Lens CS2308ZM05	1	16.99	16.99	link SKU: LN038	Other
Item 43	PureThermal Mini Pro JST-SR (with FLIR Lepton 3.5)	1	369.95	369.95	link SKU: DEV-17544	Other
Item 44	USB Type-A to Type-B cable	1	4	4	Local hardware store	Other
Item 45	1 Kg PLA filament	2	14	28	link ASIN: B07XG3RM58	Polymer
Item 46	Terminal strip	1	1	1	linkASIN: B08V4W637Q	Polymer

The reported cost corresponds to the component prices at the time of device construction.

Supplies for soldering, hot glue, tools, and labor are not included in this budget quotation.

The reported cost corresponds to the component prices at the time of device construction.

Supplies for soldering, hot glue, tools, and labor are not included in this budget quotation.

## 5 Build instructions

1. General assembly of the device

The construction of the device involved three main stages: (i) assembly of the mechanical support structure, (ii) completion of the electrical connections between components, and (iii) programming of the control software for system operation.

2. Construction of the main structure

A rectangular parallelepiped was built using square iron pipes (items 1, 2, and 3), which were welded together to form the supporting structure (Fig. 4a).

3. Structural reinforcement using tensioners

**Fig. 4:**
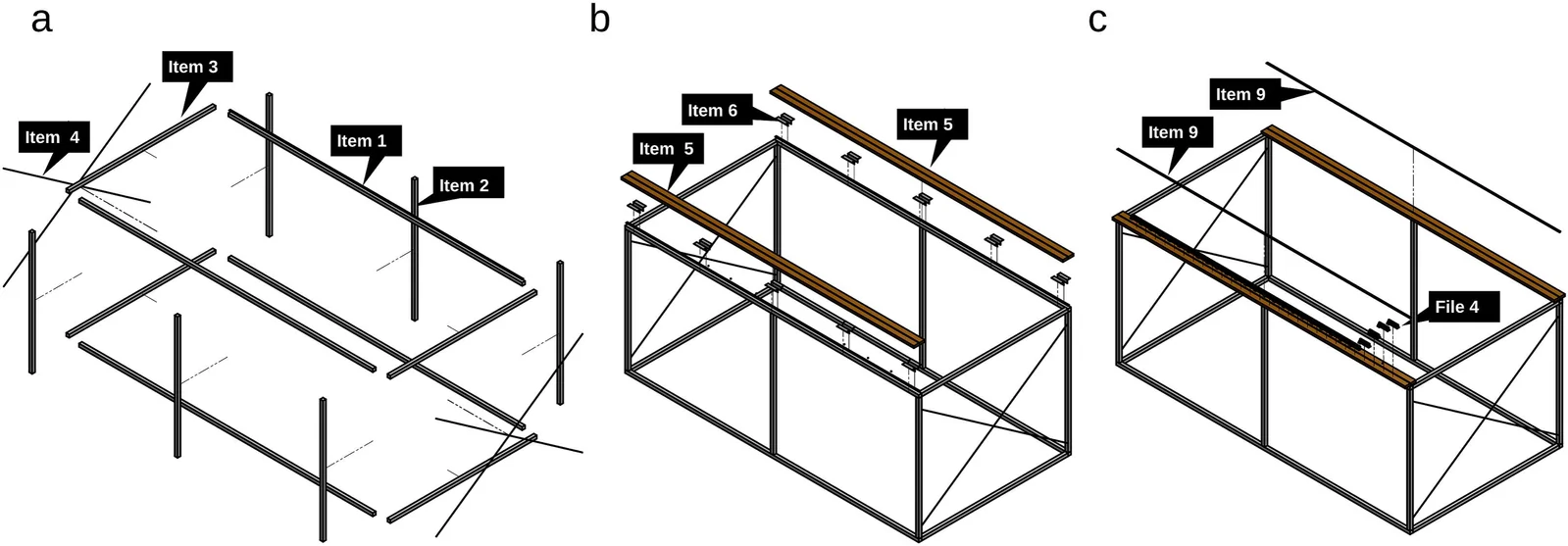
Successive stages in the assembly of the main structure of the phenotyping platform. (a) Welded frame built from square steel tubes, with the crossed wires that stiffen the front and rear faces. (b) Installation of the wooden boards forming the upper bases of the x-axis guidance system, fastened to the frame with L-shaped aluminum angles. (c) Mounting of the aluminum guide rails on both boards and of the 3D-printed rack on one of them. Item 1, Item 2 and Item 3, square steel tubes of the frame. Item 4, tensioning wires. Item 5, wooden boards. Item 6, aluminum angles. Item 9, aluminum guide rails. File 4, rack segments (file4_long_rack.stl). Item numbers correspond to the bill of materials, and file numbers to the design files summary.

To increase the mechanical rigidity of the structure, crossed tensioning wires (item 4) were incorporated on the front and rear faces of the parallelepiped (Fig. 4a).

4. Installation of upper bases for the guidance system (x axis)

Two wooden boards (item 5) were placed on the long edges of the upper face of the structure, one on each side, which were secured with L-shaped aluminum angles (item 6), rivets (item 7), and screws (item 8) (Fig. 4b).

5. Mounting of the macro-carriage guide rails (x axis)

An aluminum rail (item 9) was installed on each of the boards, which acts as a guide for the macro-carriage during its displacement along the x axis (Fig. 4c).

6. Installation of the x-axis rack

On one of the lateral boards, a rack composed of 14 3D-printed segments (file4_long_rack.stl) was installed, intended to interact with the pinion (file3_pinion.stl) of the axis traction mechanism (Fig. 4c). Wood screws (item 10) were used to secure the rack to the board.

7. Use of the alignment guide for rack segments

To facilitate the assembly and alignment of the 3D-printed rack segments, it is recommended to use the designed positioning guide (file5_long_rack_guide.stl), as shown in Fig. 5.

8. Placement of x-axis reference magnets

**Fig. 5:**
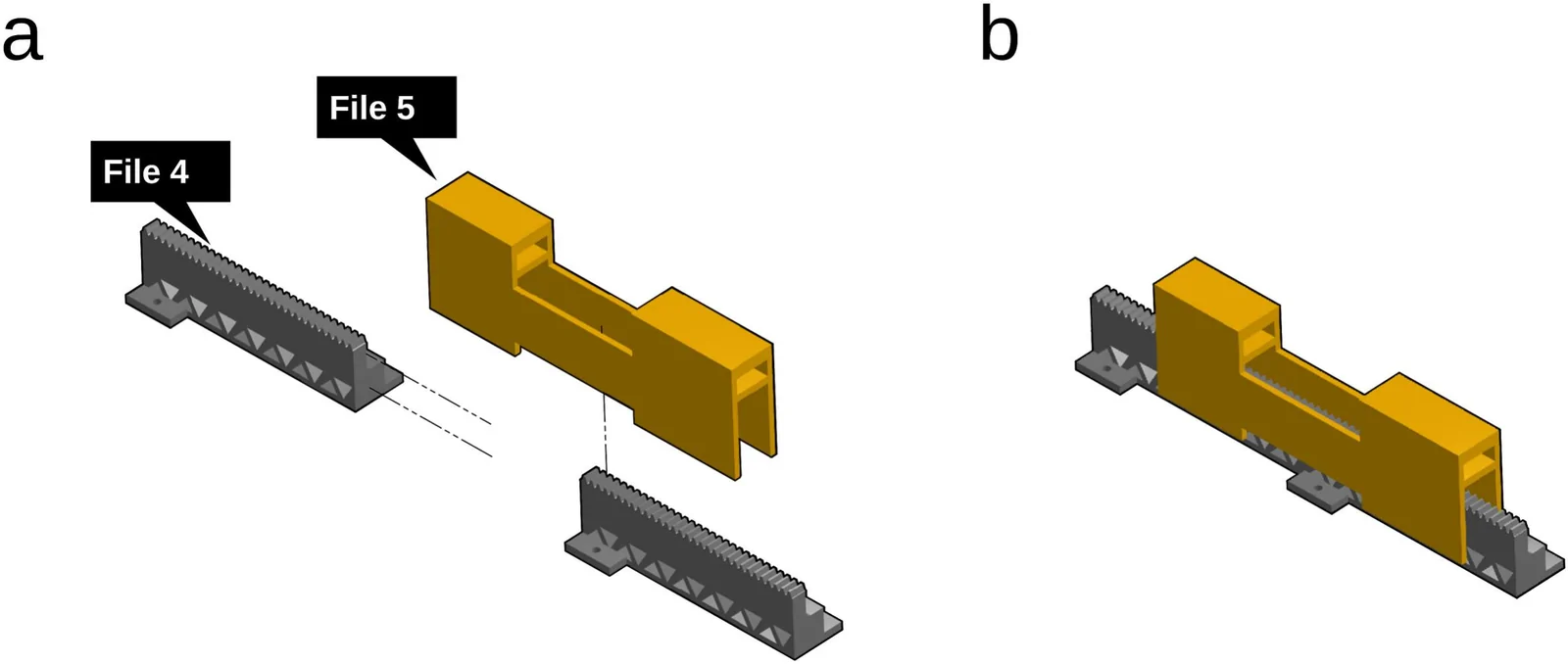
Use of the alignment guide for the correct positioning of the 3D-printed rack segments during their installation. (a) Rack segments before insertion into the guide. (b) Rack segments fully seated in the guide, which sets the spacing between consecutive segments so that the tooth pitch remains continuous across the joint. File 4, rack segment (file4_long_rack.stl). File 5, alignment guide (file5_long_rack_guide.stl).

Seven magnets (item 11) were placed on the right lateral board, positioned along the x axis at coordinates corresponding to the intended location of the plants (Fig. 3). These magnets are used as discrete position references for the Hall effect sensing system.

9. Construction of the macro-carriage (x-axis mobile structure)

The macro-carriage was built using two wooden pieces (items 12 and 13), joined by two L-shaped aluminum angles (item 14) secured at their ends with screws, nuts, and washers (items 15, 16, and 17). To prevent bowing of items 12 and 13, they were reinforced by placing L-shaped aluminum angles (item 18) on their underside, secured with wood screws (item 8) (Fig. 6).

10. Mounting of the macro-carriage guide wheels (same procedure for the micro-carriage)

**Fig. 6:**
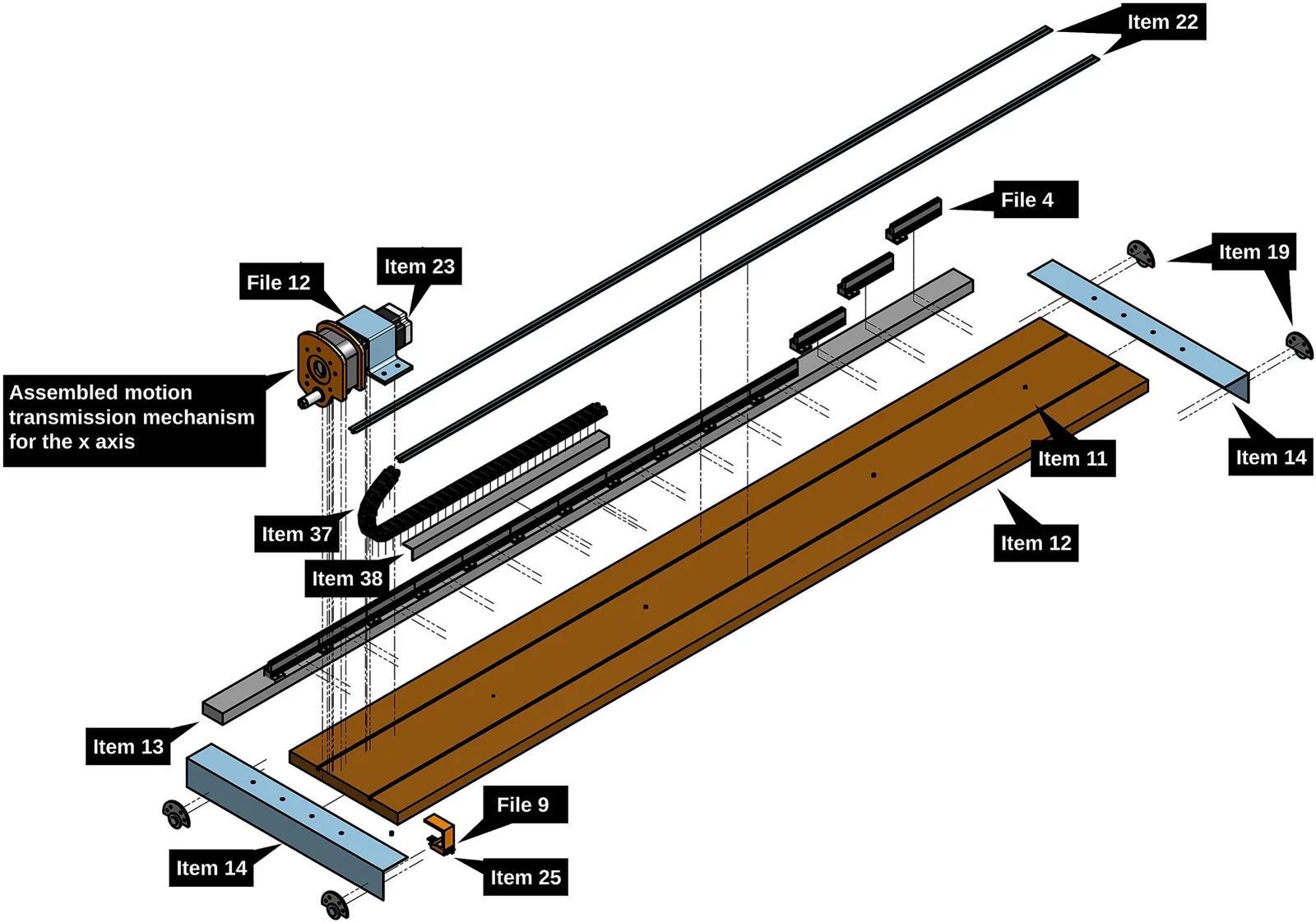
Exploded view of the macro-carriage assembly. Item 11, magnets marking the discrete positions along the y axis. Item 12, wide wooden board. Item 13, narrow wooden board carrying the y-axis rack. Item 14, L-shaped aluminum angles joining both boards at their ends. Item 19, roller sets acting as guide wheels on the x-axis rails. Item 22, aluminum guide rails for the displacement of the micro-carriage along the y axis. Item 23, NEMA23 stepper motor for the x-axis movement. Item 25, Hall effect sensor module. Item 37, cable drag chain. Item 38, L-shaped aluminum angle supporting the cable drag chain, fixed to the narrow board. File 4, rack segments (file4_long_rack.stl) forming the y-axis rack. File 9, Hall effect sensor support (file9_Hall_sensor_support.stl). File 12, motor mounting bracket for the x axis (file12_support_motor_x.stl). The assembled motion transmission mechanism for the x axis is indicated as a unit; its components are detailed in Fig. 8. Item numbers correspond to the bill of materials, and file numbers to the design files summary.

Four wheels (item 19) were installed on the sides of the L-shaped aluminum angles. These wheels were secured using bolts (item 20) and wing nuts (item 21), allowing fine adjustment of their position to ensure proper fitting onto the aluminum rails (Fig. 6).

11. Installation of the y-axis rack

On the narrow wooden piece of the macro-carriage (item 13), a rack composed of seven segments (file4_long_rack.stl) was installed, intended to interact with the pinion (file3_pinion.stl) of the y-axis traction mechanism (Fig. 6).

12. Mounting of the y-axis rails on the macro-carriage

Two aluminum rails (item 22) were placed on the wide wooden piece (item 12), as shown in Fig. 7. These rails allow the displacement of the micro-carriage along the y axis.

13. Placement of y-axis reference magnets

**Fig. 7:**
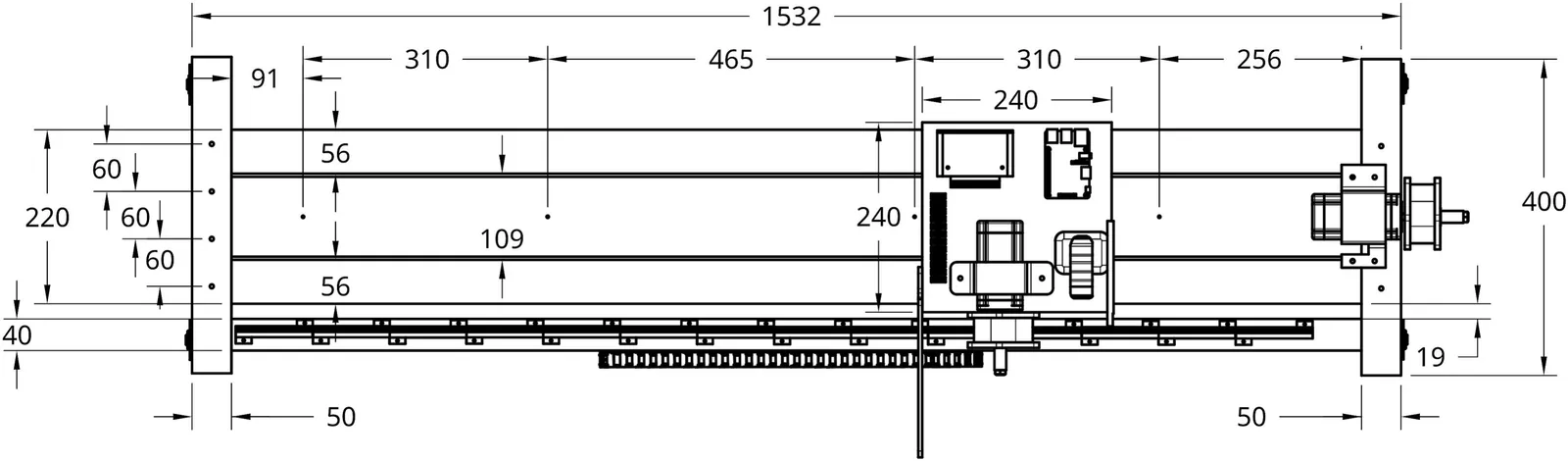
Structural detail of the macro-carriage. 1, support for guiding the cables coming from the micro-carriage. 2, magnets indicating the stopping positions on the y axis. 3, holder and motor for movement along the y axis. 4, holder and motor for movement along the x axis. 5, driver for the y-axis movement motor. 6, Raspberry Pi board. 7, electrical outlet and adapter for Raspberry Pi power supply. 8, on the underside of this end, the Hall sensor for sensing the positioning magnets on the x axis is located. Dimensions are expressed in millimeters.

Four magnets (item 11) were placed on the wide wooden piece of the macro-carriage, at positions defined according to Fig. 7. These magnets correspond to discrete coordinates on the y axis, determined by the intended location of the plants.

14. Considerations for magnet orientation with a unipolar Hall sensor

Because the Hall effect sensor used is unipolar, it only detects one pole of the magnet. Therefore, during assembly, the detectable pole must be oriented toward the Hall sensor to ensure consistent position reading.

15. Mounting of the x-axis motor and its traction system

A NEMA23 stepper motor (item 23) was installed on the right side of the macro-carriage, together with the pinion (file3_pinion.stl) and the 3D-printed traction mechanism (file2_pulley_8m_nema23.stl), responsible for the displacement of the macro-carriage along the x axis.

16. Installation of the x-axis electronics on the underside of the macro-carriage

On the underside of the macro-carriage, the following were installed: (i) the x-axis stepper motor controller unit (item 24) and (ii) a Hall effect sensor module (item 25). A 3D-printed adapter (file9_Hall_sensor_support.stl) was used to mount this module. This adapter is screwed to the micro-carriage, and the sensor module is inserted into the support and secured with hot glue.

17. Critical alignment of the x-axis Hall sensor

The Hall sensor must be correctly aligned with the magnets arranged along the x axis, as these signals determine both the x coordinates of the plants and the resting position of the macro-carriage.

18. Assembly of the motion transmission mechanism for each axis

The traction mechanism (Fig. 8) is composed of 3D-printed parts (file3_pinion.stl, file2_pulley_8m_nema23.stl, file4_long_rack.stl), two bearings (item 26), a transmission belt (item 27), a bolt (item 28), and a self-locking nut (item 29). The traction mechanism components were assembled and secured with threaded rods and nuts (items 30 and 31), maintaining the mechanical integrity of the assembly. One such mechanism was assembled for each motor, corresponding to each of the movement axes (x, y).

19. Adjustment of the pinion–rack gear (x axis)

**Fig. 8:**
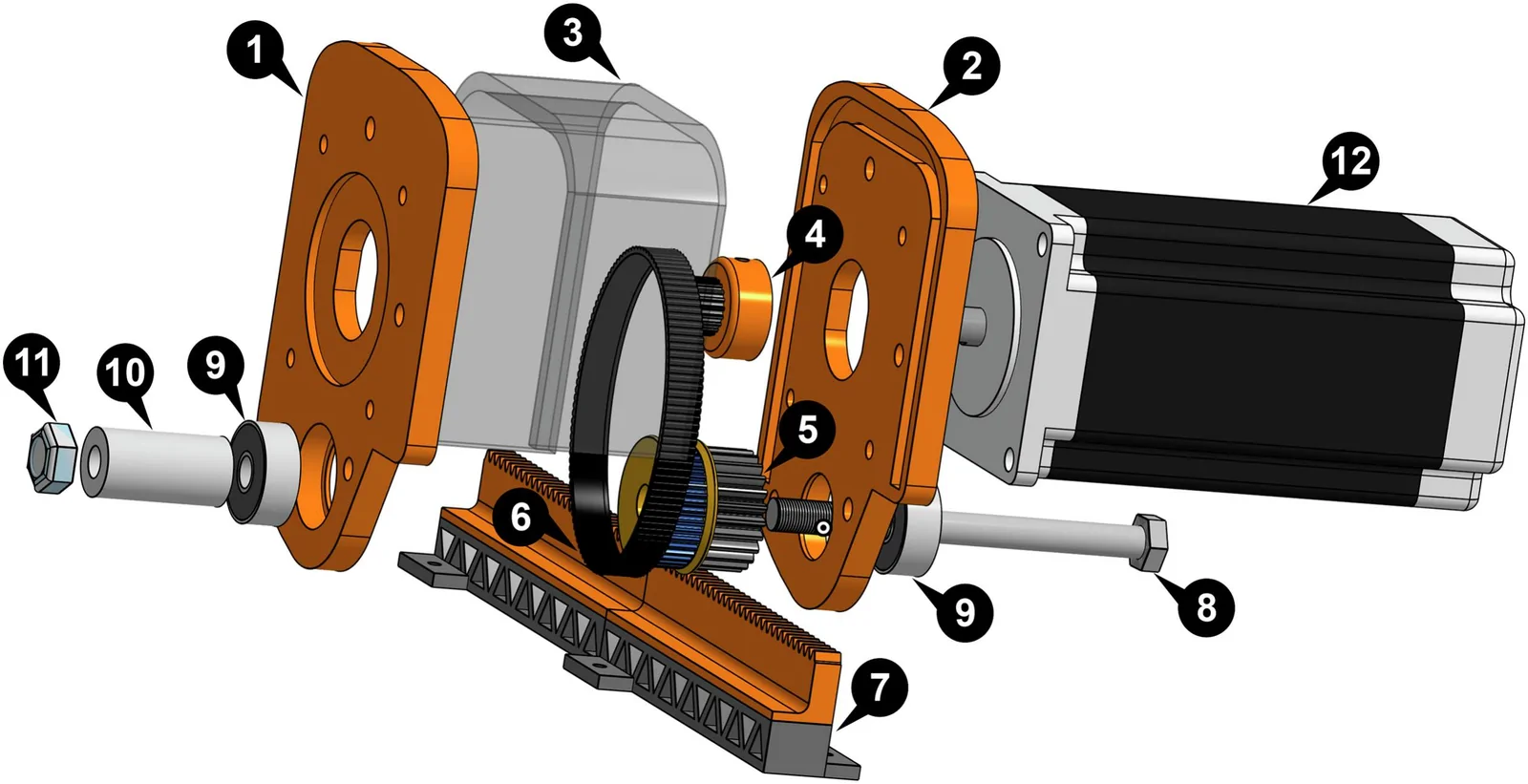
Exploded schematic view of the assembled motion transmission mechanism for each axis. 1, 2, front (file1b_cover_motor_front.stl) and rear (file1c_cover_motor_back.stl) parts of the transmission cover. 3, intermediate piece (file1a_cover_motor_bridge.stl) of the transmission cover. 4, transmission pulley (file2_pulley_8m_nema23.stl). 5, traction pulley and pinion (file3_pinion.stl). 6, motion transmission belt (item 27). 7, rack (file4_long_rack.stl). 8, bolt (item 28). 9, bearing (item 26). 10, adjustment adapter (file8_cylindrical_adapter.stl). 11, self-locking nut (item 29). 12, NEMA23 motor (item 23).

Prior to placing the motor mounting bracket (file12_support_motor_x.stl), it must be positioned so that the pinion, installed on the macro-carriage, makes adequate physical contact with the rack installed on the main structure. This contact is critical to prevent step losses, slippage, or positioning failures.

20. Mounting of the micro-carriage (y-axis mobile structure)

The micro-carriage, responsible for movement along the y axis, was installed on the macro-carriage. The micro-carriage base was built from a 24 cm square board (item 32) (Fig. 9).

21. Installation of the micro-carriage lower profiles

**Fig. 9:**
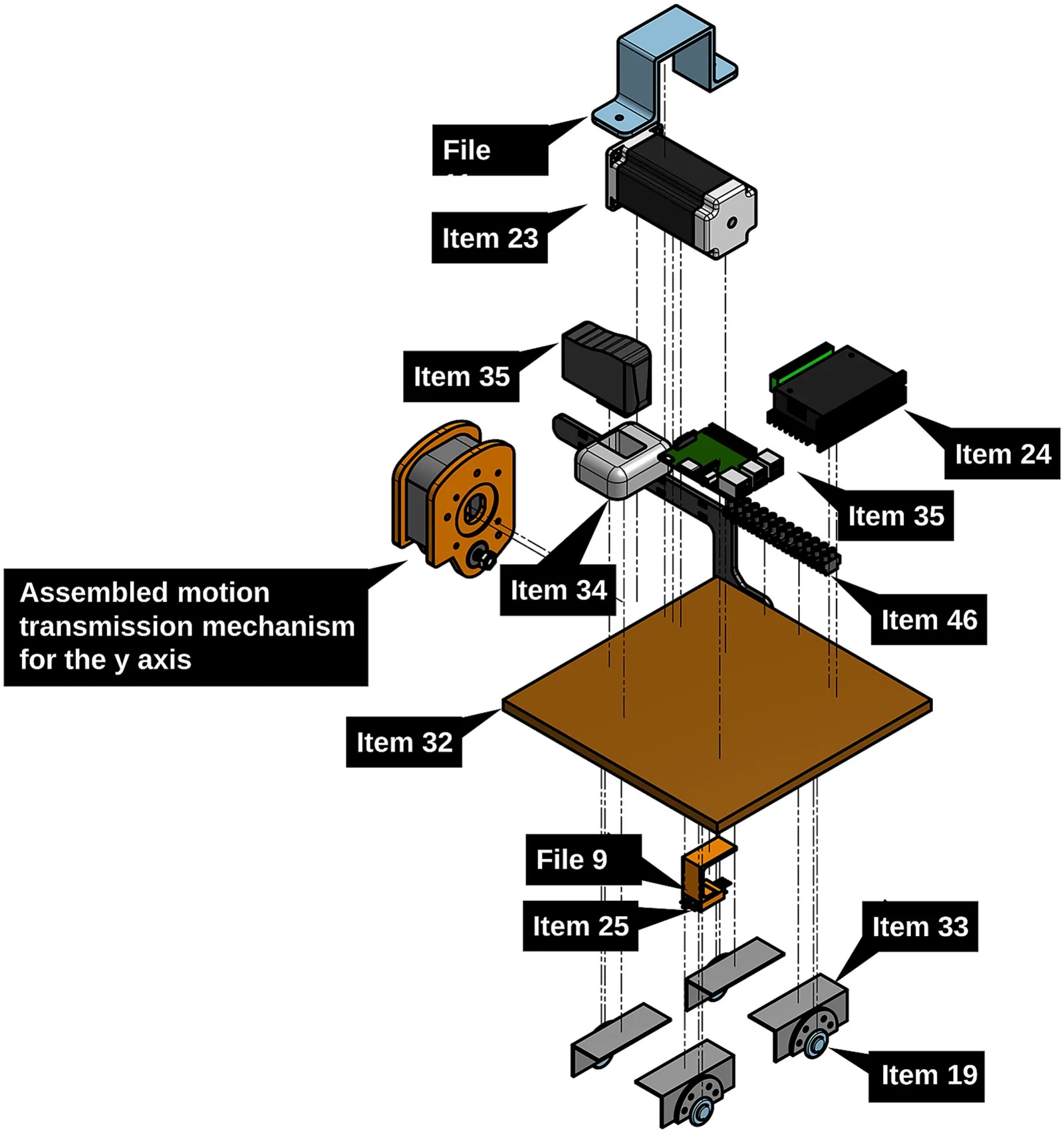
Exploded view of the micro-carriage assembly. Item 19, roller sets acting as guide wheels on the y-axis rails. Item 23, NEMA23 stepper motor for the y-axis movement. Item 24, stepper motor driver. Item 25, Hall effect sensor module, mounted on the underside of the base. Item 32, square wooden board forming the micro-carriage base. Item 33, L-shaped aluminum angles secured underneath the base as supports for the wheels. Item 34, power outlet. Item 35, Raspberry Pi 3B + board and its electrical adapter, both supplied in the same kit. Item 46, terminal strip. File 9, Hall effect sensor support (file9_Hall_sensor_support.stl). File 11, motor mounting bracket for the y axis (file11_support_motor_y.stl). The assembled motion transmission mechanism for the y axis is indicated as a unit; its components are detailed in Fig. 8. Item numbers correspond to the bill of materials, and file numbers to the design files summary.

Four L-shaped aluminum angles (item 33) were secured underneath the micro-carriage base, which act as supports for the wheel system (item 19) (Fig. 9).

22. Mounting of the micro-carriage wheels and adjustment onto the y-axis rails

A wheel was placed on the side of each profile, defining an appropriate spacing between wheels to fit onto the macro-carriage rails and allow controlled displacement along the y axis.

23. Installation of the y-axis Hall sensor

A Hall effect sensor module (item 25) was installed on the underside of the micro-carriage to detect the magnets (item 11) indicating the y-axis coordinates associated with each position. The y-axis Hall sensor must be carefully aligned with the magnets fixed on the upper part of the macro-carriage (Fig. 10), ensuring repeatable readings and precise determination of the micro-carriage position. A support similar to the one used for the x-axis Hall sensor (file9_Hall_sensor_support.stl) is also used for this sensor.

24. Installation of electronics on the micro-carriage

**Fig. 10:**
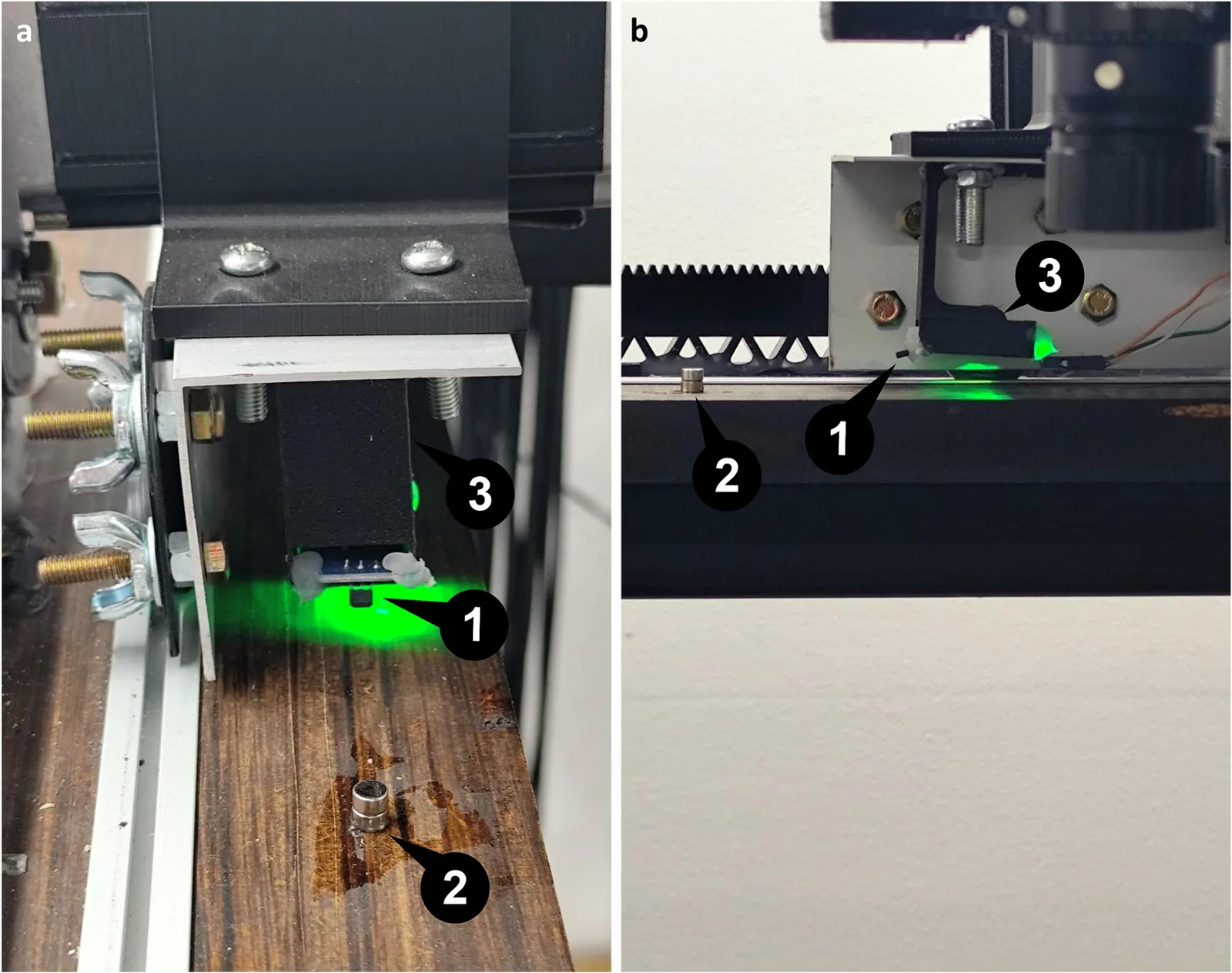
Detail of the Hall effect sensor installed under the micro-carriage for sensing the marked positions along the y axis using magnets. (a) Front view. (b) Side view. 1, Hall effect sensor. 2, Magnets placed aligned with the trajectory along which the sensor travels. 3, sensor support (file9_Hall_sensor_support.stl).

A power outlet (item 34) was installed on the upper part of the micro-carriage, used to power the Raspberry Pi (item 35) board through its electrical adapter (Fig. 9).

25. Installation of the y-axis motor and its motion transmission mechanism

The following were installed on the micro-carriage: (i) a NEMA23 stepper motor (item 23), (ii) the pinion (file3_pinion.stl), and (iii) the traction mechanism responsible for movement along the y axis (file2_pulley_8m_nema23.stl, file4_long_rack.stl). Similarly to the x-axis motor mounting, care must also be taken here to establish proper contact between the pinion and the rack prior to placing the y-axis motor mounting bracket (file11_support_motor_y.stl). The driver unit (item 24) corresponding to this motor was also placed on the micro-carriage. The layout of the components installed on the micro-carriage and their location are shown in Fig. 11.

26. Installation of the terminal strip on the micro-carriage

**Fig. 11:**
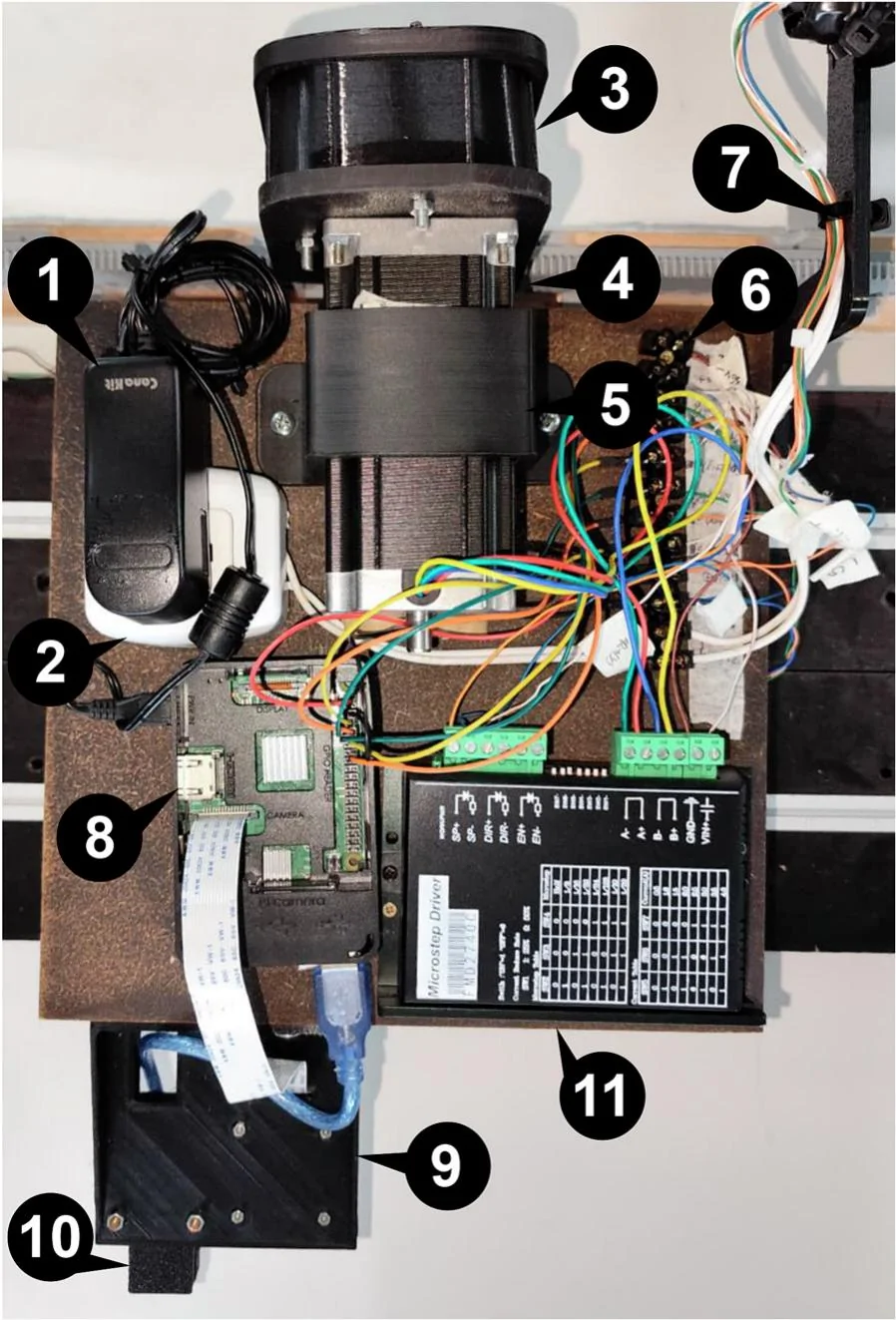
Location of the components on the micro-carriage. 1, electrical adapter. 2, power outlet (item 34). 3, transmission cover. 4, NEMA23 motor (item 23) attached to the base by the 3D-printed holder. 5, support for the y-axis motor mounting. 6, terminal strip for electrical connections. 7, cable support for the micro-carriage. 8, Raspberry Pi 3B + board (item 35). 9, camera head. 10, cover for the thermographic module. 11, NEMA23 motor driver (item 24).

A terminal strip (item 46) was installed on the upper surface of the micro-carriage, as shown in Fig. 11 (element 6). This component serves as the central wiring node for all electrical connections on the micro-carriage, including the signal and power lines between the Raspberry Pi board, the two stepper motor drivers (item 24), and the Hall effect sensor modules (item 25). Organizing all connections through a single terminal strip simplifies assembly, facilitates troubleshooting, and allows individual components to be disconnected or replaced without re-soldering. The terminal strip was secured to the micro-carriage base using wood screws (item 10).

27. General system wiring and power architecture

The wiring diagram for the connection of the components is shown in Fig. 12. The device operates with two independent power circuits, both originating from a 220 V AC wall socket located outside the growth chamber.

- Circuit 1 (220 V AC): a mains cable with plug runs from the wall socket along the underside of one of the lateral wooden boards (item 5), routed with sufficient slack to accommodate the full travel of the macro-carriage along the x axis. The cable continues under the macro-carriage to the terminal strip (item 46), and from there it is routed through the cable carrier chain (item 37) to the power outlet (item 34) installed on the micro-carriage. The Raspberry Pi power adapter plugs into this outlet, and the Hall effect sensor modules are powered from the Raspberry Pi GPIO header.
- Circuit 2 (36 V DC): a 36 V, 11 A DC power supply (item 36) is plugged into a separate 220 V AC wall socket and is placed at a fixed position near the base of the platform frame. Its DC output cables run alongside the 220 V AC line along the same path (under the lateral board, through the macro-carriage, and through the cable carrier chain) to reach the two stepper motor driver units (item 24), one located on the macro-carriage and one on the micro-carriage.
28. Management of mobile cabling using a cable carrier chain

**Fig. 12:**
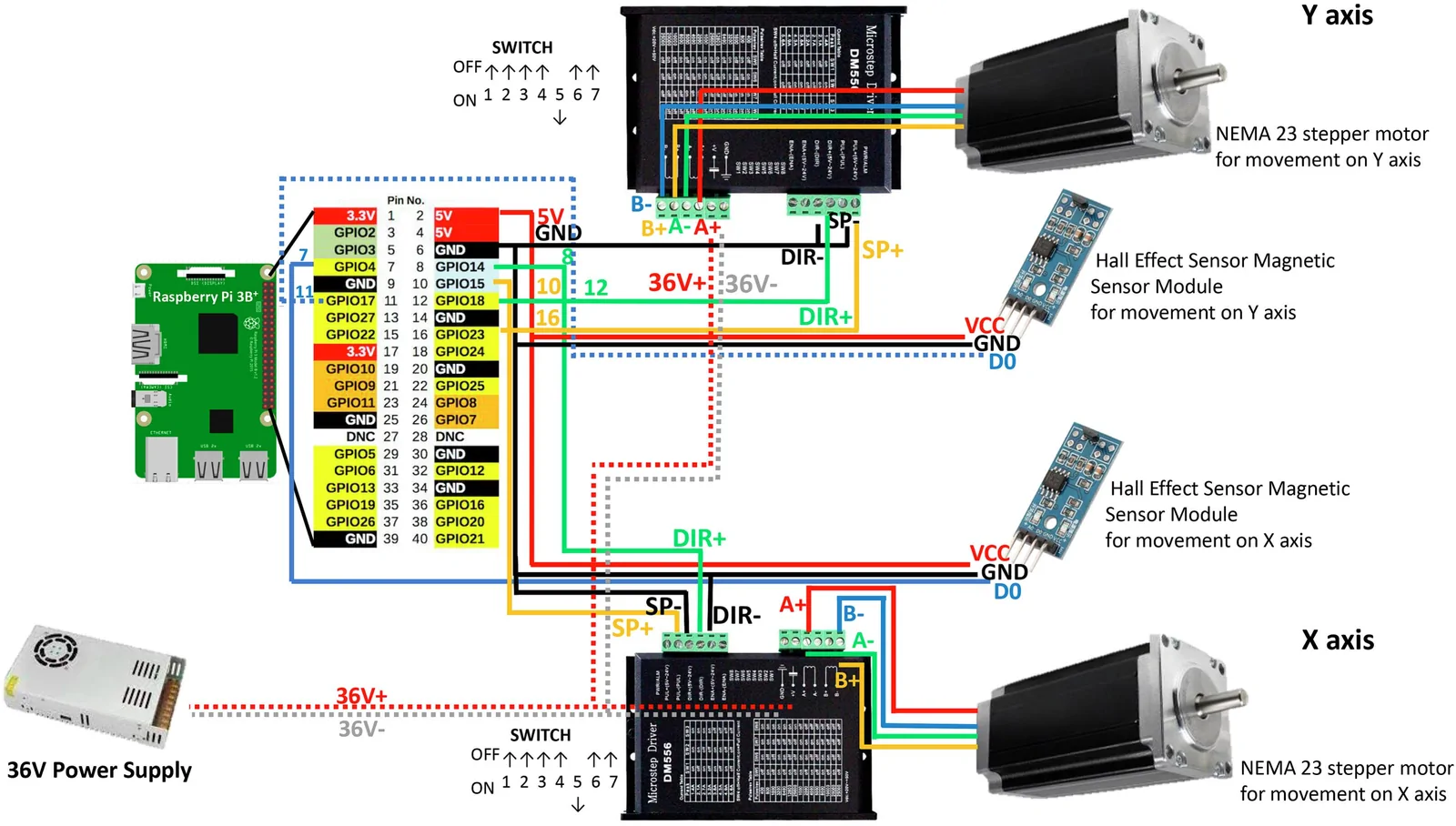
Electrical connections diagram. The Raspberry Pi 3B + pins are colored accordingly: Red, 5 V or 3.3 V power supply. Black, ground. Yellow, General Purpose Input/Output pins. Gray, reserved pins. Light blue, green, and orange pins for special functions. (For interpretation of the references to colour in this figure legend, the reader is referred to the web version of this article.)

To reduce mechanical stress and organize the cables associated with the micro-carriage (cables with one end in motion), a Cable Wire Carrier Drag Chain (item 37) was used, as shown in Fig. 3, secured to an L-shaped aluminum angle (item 38).

29. Installation of the RGB camera (Raspberry Pi HQ Camera) and optical configuration

A 3D-printed support (file7_cameras_support.stl, Fig. 13) was designed and installed, secured to the underside of the micro-carriage base. Underneath this support, a Raspberry Pi High Quality Camera (HQ Camera) (item 39) was mounted, secured with bolts and nuts (items 40 and 41). The camera position is shown in Fig. 13. The camera was connected to the CSI-2 (MIPI CSI-2) camera port of the Raspberry Pi. Additionally, this camera allows the use of interchangeable lenses with different optical properties. In the configuration used in this device, the Arducam HQ Lens model CS2308ZM05 (item 42) was employed, selected based on the required field of view and working distance, from the variety of lenses offered for this camera.

30. Installation and connection of the thermographic camera (PureThermal board + FLIR Lepton 3.5 sensor)

**Fig. 13:**
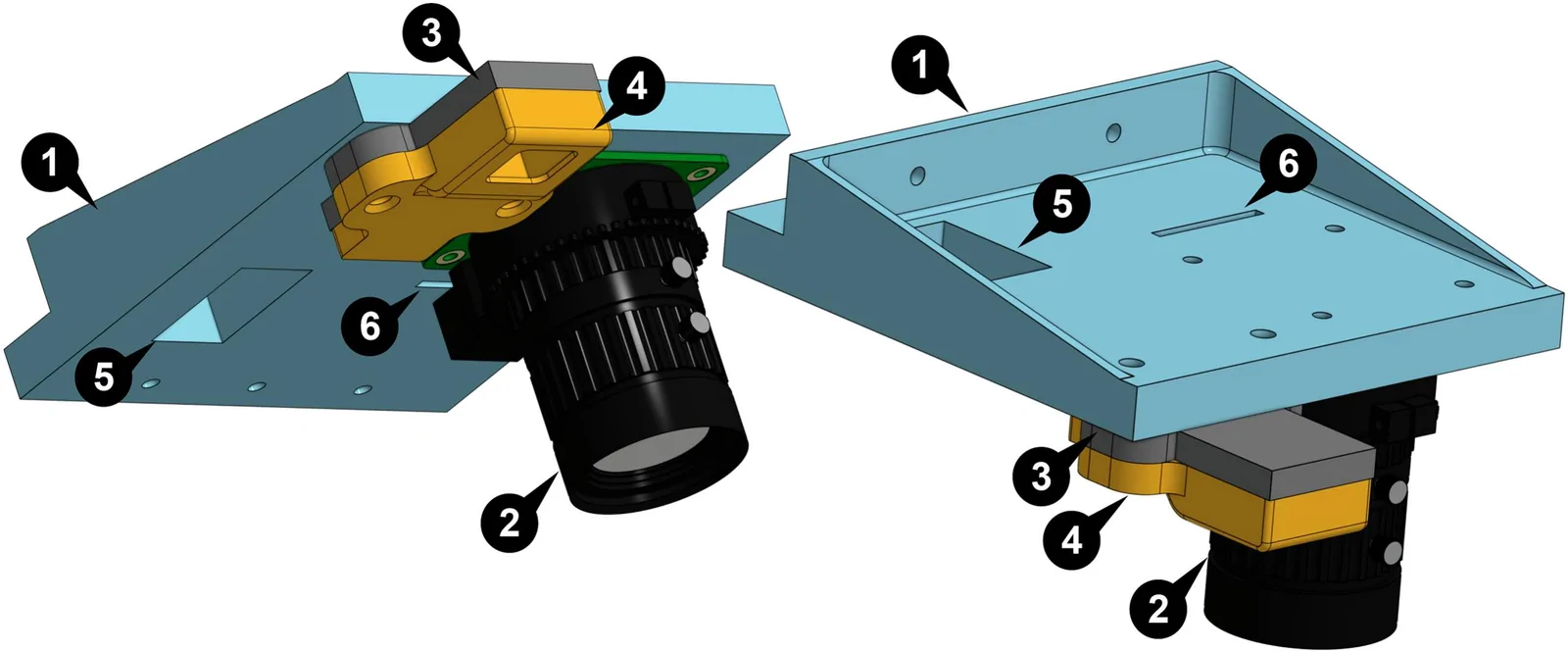
Support for the RGB camera and the thermographic module. The Raspberry Pi HQ Camera is installed directly on the underside of the support, and the thermographic module is placed inside the 3D-printed housing. 1, support (file7_cameras_support.stl). 2, RGB camera. 3, 4, front (file6_thermal_cover_front.stl) and rear (file6_thermal_cover_back.stl) parts of the housing containing the thermographic module. 5, slot for the passage of the thermographic module USB cable. 6, slot for the passage of the RGB camera CSI cable.

A PureThermal Mini Pro JST-SR board equipped with a FLIR Lepton 3.5 thermographic sensor (item 43) was installed on the previously described support, as shown in Fig. 13. The PureThermal board was placed inside a 3D-printed adapter (file6_thermal_cover_front.stl, file6_thermal_cover_back.stl) to ensure its fixation and mechanical alignment. The module was then connected to the Raspberry Pi via a USB interface (UVC-type device), using a USB cable (item 44) soldered at one end to the terminals/pads of the board according to the manufacturer’s specifications [31]. Instead of the standard JST-SR cable, a disassembled USB cable was used, connected directly to the through holes of the board (Fig. 14). The connections are indicated in Table 4.

31. Installation of the cover on the micro-carriage (optional)

**Fig. 14:**
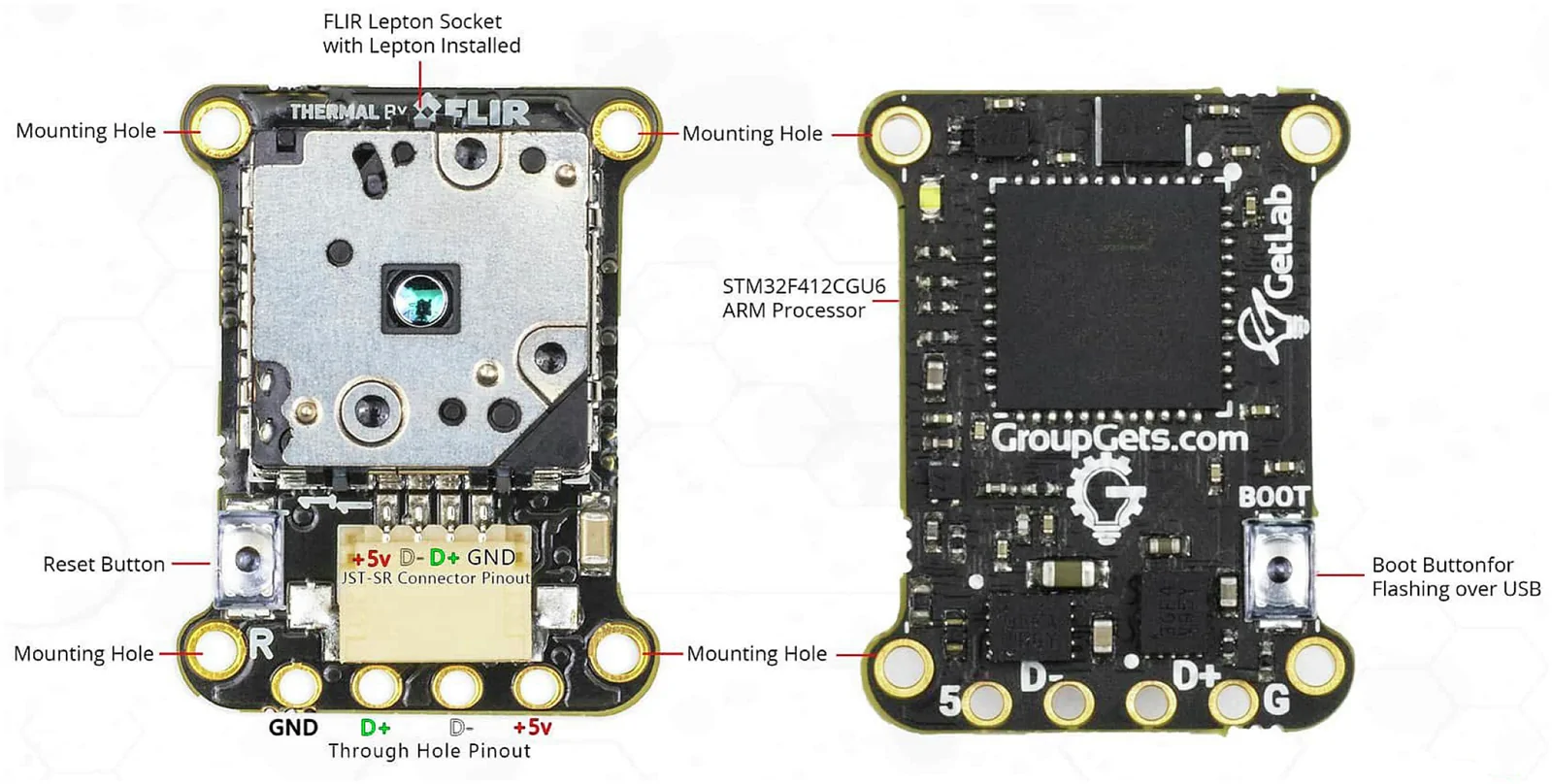
Terminal diagram for the PureThermal Mini Pro JST-SR board connection.

Adapted from [31].

**Table 4:** Connection diagram of the PureThermal Mini Pro JST-SR board to the USB cable.

**USB Cable**	**Standard Color**	**PTM Terminal**
+5V	Red	+5v
GND	Black	GND
D+	Green	D+
D-	White	D-

If you wish to protect the micro-carriage with a cover, attach the cover base (3D-printed from file10a_main_cover.stl) to the micro-carriage using four wood screws (item 8), and then place the cover (3D-printed from file10b_main_cover.stl) on top.

Note: For all 3D prints involved in this work, an FDM 3D printer (Artillery Sidewinder X1 model) was used with PLA filament (item 45).

## 6 Operation instructions

### 6.1 Local operation

1. Place the pots with plants in the 24 available positions, defined by the x and y coordinates of the magnets located on each axis of the platform. The height at which the plants are placed will depend on their size at the developmental stage of interest.
2. Manually adjust the focus of the RGB camera and use an appropriate lens for the distance at which the plants are located.
3. Connect the platform to the electrical power supply by plugging both cables into their respective 220 V AC wall sockets: one for the Raspberry Pi power line and one for the 36 V DC power supply (see step 27 for details).
4. If you have not configured automatic internet connection and remote access, you will need to connect a keyboard, mouse, and monitor to the platform’s Raspberry Pi board.
5. Navigate to the directory where the Python files downloaded from the repository associated with this article are located.
6. Run the main program main_gui.py with the command: python3 main_gui.py. This will display the graphical user interface (Fig. 15) through which you can control the platform.

**Fig. 15:**
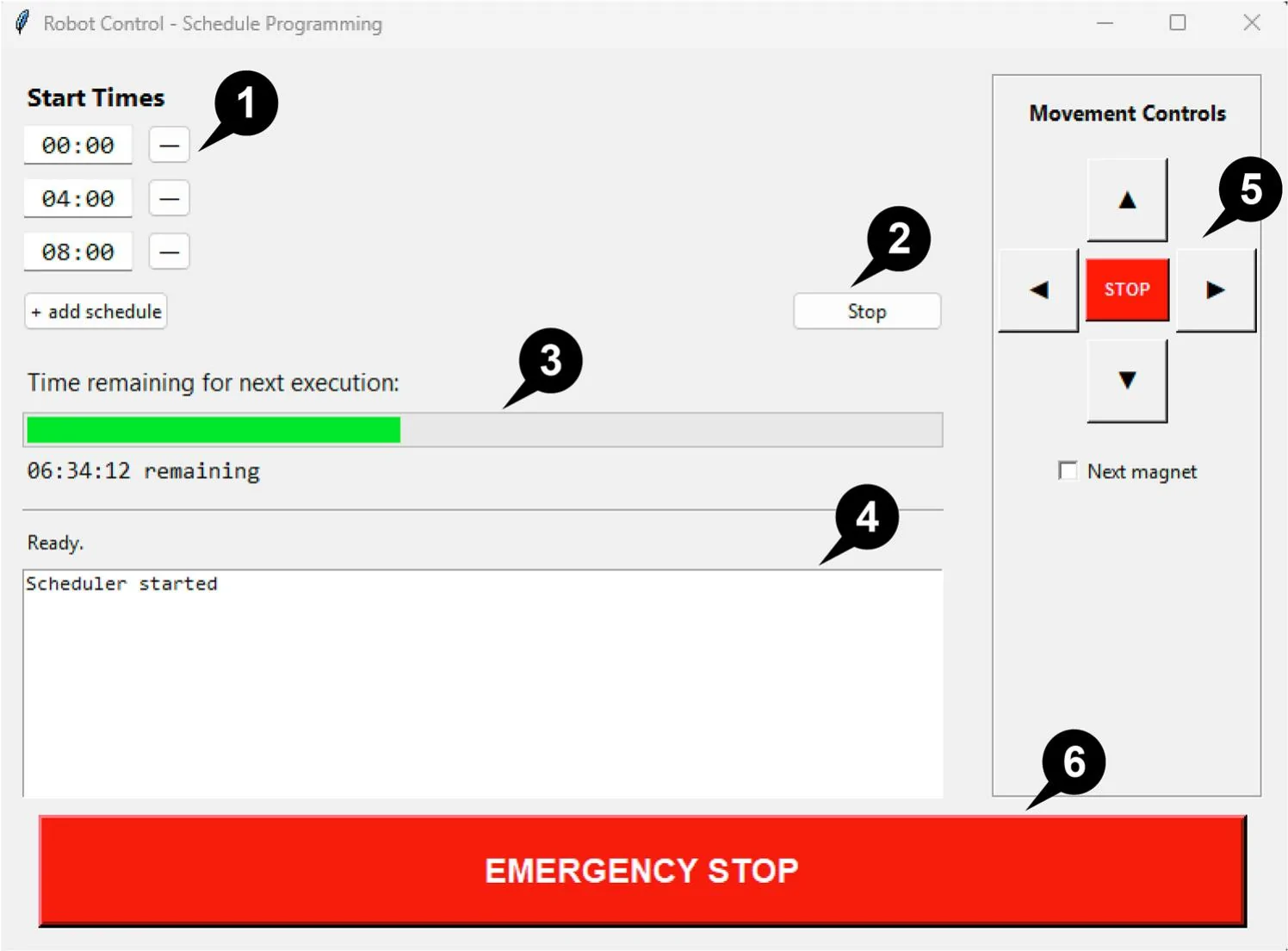
Graphical user interface for operating the open phenotyping platform on a Raspberry Pi graphical desktop. 1, start times for the RGB and thermal image acquisition routine. 2, button to start or stop program execution. 3, progress bar showing the remaining time until the next execution of the image acquisition routine. 4, status log displaying messages about the current state of the platform during its operation. 5, section of the interface dedicated to manual movement of the macro-carriage (in the x-axis direction) and the micro-carriage (in the y-axis direction). Optionally, by activating the “Next magnet” checkbox, both carriages can be operated to move discretely between each contiguous position marked by the magnets located on both axes. 6, emergency stop button that immediately halts all platform operations at any moment, overriding any ongoing routine or movement command.

**Fig. 16:**
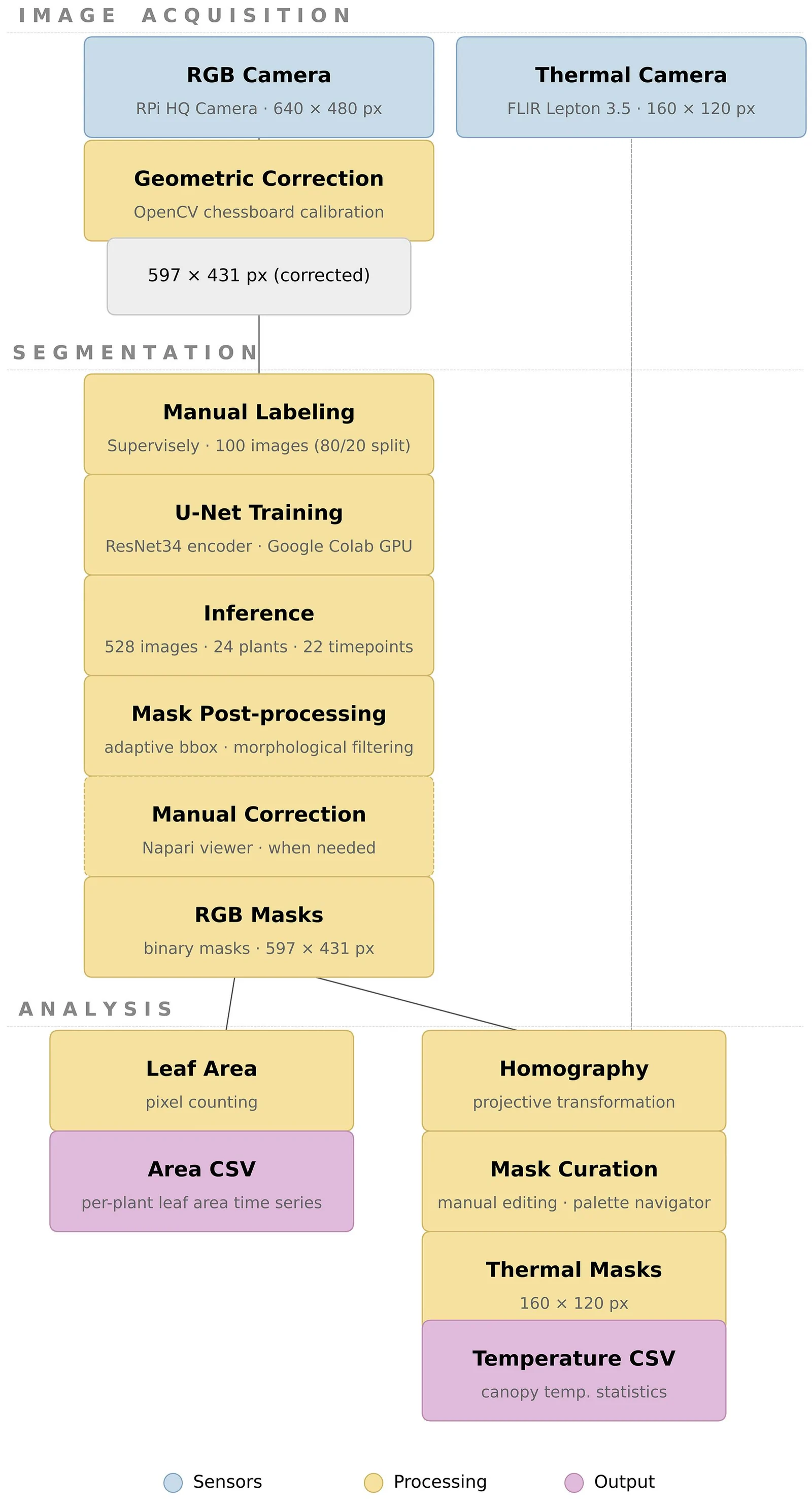
Image processing workflow for plant phenotyping. RGB images (640 × 480 px) are geometrically corrected using OpenCV calibration parameters, then segmented using a U-Net deep learning model trained on 100 manually labeled images in Supervisely. The resulting masks (597 × 431 px) are used for leaf area quantification (pixel counting) and, after homography projection to thermal resolution (160 × 120 px), as initial references for the manual curation of plant masks on the thermal images, from which canopy temperature statistics are extracted.

**Fig. 17:**
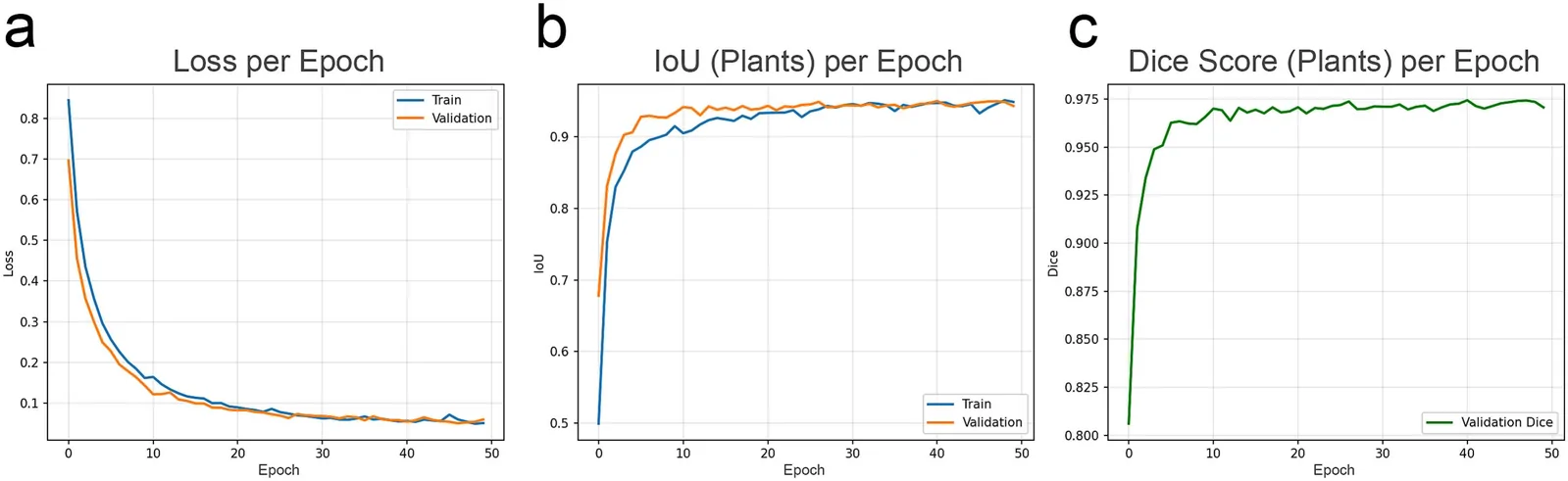
Training performance of the U-Net segmentation model over 50 epochs. (a) Training and validation loss curves showing convergence without overfitting. (b) Intersection over Union (IoU) for the plant class on training and validation sets. (c) Dice coefficient on the validation set. Final validation metrics reached IoU = 0.95 and Dice = 0.975, indicating accurate segmentation of plant tissue from RGB images acquired by the platform.

**Fig. 18:**
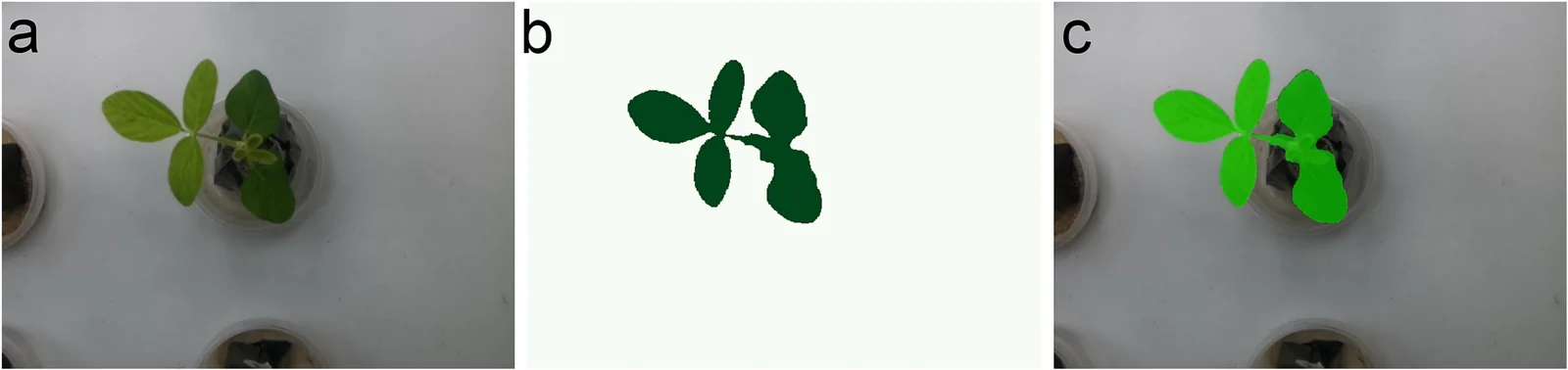
Example of plant segmentation from an RGB image acquired by the platform. (a) Original image showing a soybean plant in the central position. (b) Binary mask predicted by the U-Net model. (c) Overlay of the predicted mask on the original image. The model accurately delineates leaf boundaries and separates the target plant from the background, including the substrate, pot, and neighboring plants.
7. In case the macro- and micro-carriage are not in their resting positions, you can move them manually using the directional button pad of the interface (point 5, Fig. 15). Optionally, this manual control also allows moving the carriages discretely, between contiguous magnets, when the “Next magnet” checkbox is activated.
8. Configure the schedules to start the image acquisition routine throughout the day, using the controls indicated by point 1 of Fig. 15.
9. Start the execution of the configured program by pressing the “Start” button. Once this is done, the platform will switch to the active state, and the button will change its label to “Stop” (point 2 of Fig. 15). It should be noted that the interface clock corresponds to the Raspberry Pi system clock.
10. At any point during platform operation, the “EMERGENCY STOP” button (point 6 in Fig. 15) can be pressed to immediately halt all ongoing movements and acquisition routines. This function overrides any active command, including scheduled acquisition cycles and manual carriage displacement, bringing both motors to an immediate stop. After an emergency stop is triggered, the operator should verify that the carriages are free of obstructions before resuming operation.
11. The waiting time until the next photo acquisition routine execution is displayed on the interface along with a graphical progress bar, marked as point 3 in Fig. 15.
12. In the interface window, marked as point 4 in Fig. 15, you will be able to see messages about the current status of the platform during its operation.
13. For each of the 24 positions designated for the plants, the following files are generated in the folder **captures/**. Subfolder **picam/** containing the jpg files obtained by the RGB camera. Subfolder **lepton/** containing the files obtained by the thermographic module; in this case, for each position the following file formats are simultaneously obtained: .Y16 (raw 16-bit radiometric frame), .NPY (NumPy array), .TIFF (16-bit TIFF image), _grayscale.jpg (normalized 8-bit grayscale), _color.jpg (JET false-color mapped), and _temperature.csv (temperature matrix in °C, generated when calibrated radiometric data is available). The naming convention for all files follows the pattern <type>_X<n>_Y<m>_<timestamp>.<ext>, where n is the row (1–6) and m is the column (1–4).

A video demonstrating the complete operation sequence described in this section is available in the Mendeley Data repository (platform_operation_demo.mp4).

### 6.2 Instructions for remote operation

The platform can be operated remotely by accessing the Raspberry Pi that controls it through a virtual desktop connection. This is achieved using remote desktop software such as RealVNC (https://www.realvnc.com/), which allows visualization and interaction with the graphical user interface of the Raspberry Pi.

When the Raspberry Pi is connected to the internet through a dynamic IP address, remote access can be established using a virtual private network (VPN) based on virtual IPs, such as Tailscale (https://www.tailscale.com/). This approach enables secure remote access to the platform even when the device operates behind NAT or with an unknown external IP address.

Steps for setting up remote access:

1. Raspberry Pi setup

Install and configure the Raspberry Pi operating system and ensure network connectivity.

2. Remote desktop service (RealVNC server)

Enable and configure a remote desktop service on the Raspberry Pi using RealVNC, allowing graphical access to the system.

3. User-side remote access (RealVNC viewer)

Install a compatible RealVNC client on the user’s computer to enable remote visualization and control of the Raspberry Pi desktop.

4. Virtual private network for dynamic IP scenarios (Tailscale, optional)

When the Raspberry Pi operates under a dynamic or unknown IP address, install and configure Tailscale on both the Raspberry Pi and the user’s computer to place them within the same virtual private network.

5. Establishing the remote connection

Connect to the Raspberry Pi using RealVNC, either through the local network IP address or through the virtual IP address provided by Tailscale.

6. Remote operation of the platform

Once the connection is established, the platform can be operated remotely through the Raspberry Pi graphical interface as if it were accessed locally.

### 6.3 Safety considerations

The platform involves both mechanical and electrical hazards that users should be aware of. The two-axis Cartesian gantry includes moving carriages driven by stepper motors; operators should avoid placing hands or objects in the path of the carriages while the platform is active. The “EMERGENCY STOP” button on the graphical interface allows immediate halting of all movements. The system operates with two independent power circuits: a 220 V AC line powering the Raspberry Pi and a 36 V DC supply powering the stepper motors and drivers. All electrical connections should be performed with the power supply disconnected, and proper insulation of exposed wiring and solder joints is recommended. The platform is designed for use inside growth chambers; if the chamber includes high-humidity environments, users should ensure that all electronic components are adequately protected from condensation. Standard laboratory safety practices for working with electrical equipment should be followed at all times.

## 7 Validation and characterization

To validate the platform’s performance and demonstrate its utility for automated phenotyping, a water deficit experiment was conducted using soybean plants (*Glycine max*, cv. Genesis 5601 grown in a mix of sand:vermiculite (1:1)). Seeds were sown in 0.5 L plastic bottles (pots) and maintained in a controlled growth chamber under a photoperiod of 16 h light / 8 h dark, with day/night temperatures of 30 °C / 20 °C and a relative humidity of 35 % / 40 %, respectively. Twenty days after sowing, the 24 plants were arranged in the 6 × 4 grid of the platform in a fixed spatial configuration, with 12 plants under regular irrigation (coded R) and 12 under drought stress imposed by withholding irrigation (coded S). The complete position-to-treatment assignment is documented in the README.md file of the source file repository, under the heading “Experimental Design — Treatment Layout”, and is the assignment encoded in the analysis scripts used to produce Fig. 19, Fig. 20, Fig. 21, Fig. 22. The codes R and S are arbitrary group labels, used consistently in the image file names, the analysis scripts and the figures of this work. The platform was used to acquire RGB and thermal images at scheduled times per day over a period of 7 days, resulting in 22 acquisition sessions and a total of 528 scheduled dual-image acquisitions (24 plants × 22 timepoints). All 528 RGB images were retained for analysis; two thermal frames (X3_Y1 on 2025–09-08 09:00 and X2_Y1 on 2025–09-06 17:00) were discarded due to FLIR Lepton sensor initialization failures, leaving 526 thermal captures for canopy temperature analysis. The acquired images were then processed through two parallel pipelines: one for leaf area quantification from RGB data, and one for canopy temperature extraction from thermal data, as described below.

**Fig. 19:**
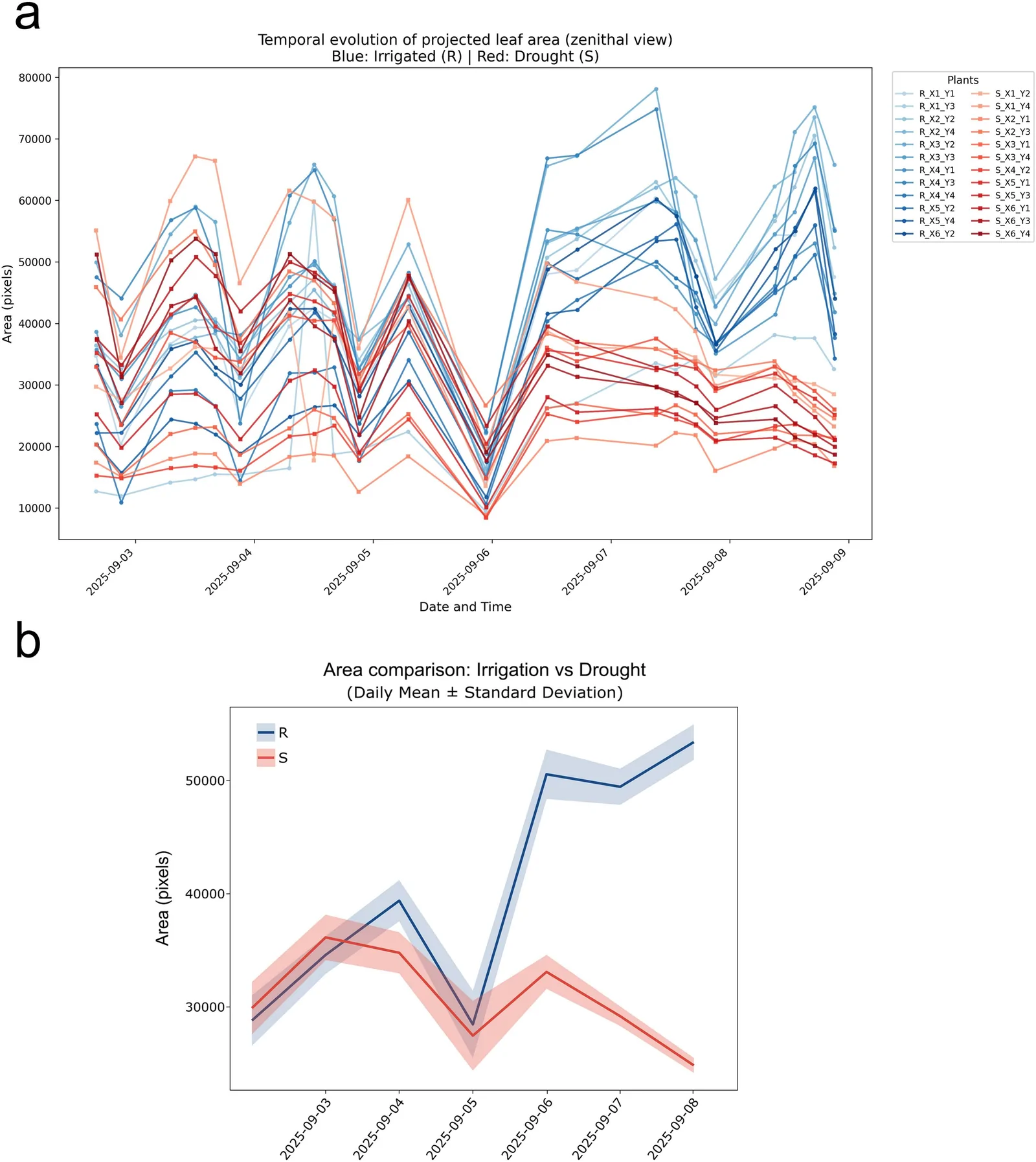
(a) Temporal evolution of projected leaf area (in pixels) for 24 individual soybean plants over a 7-day period. Blue traces correspond to irrigated plants (R, n = 12) and red traces to drought-stressed plants (S, n = 12). Irrigated plants showed sustained area expansion throughout the experiment, while drought-stressed plants exhibited a progressive decline in projected leaf area beginning approximately on day 5 (September 7), consistent with wilting and leaf curling under water deficit. The oscillatory pattern visible within each day reflects changes in leaf angle associated with the diurnal light cycle. (b) Daily mean projected leaf area (± standard deviation) for irrigated (R, blue, n = 12) and drought-stressed (S, red, n = 12) plants, showing the divergence between treatments from approximately day 5 (September 7). (For interpretation of the references to colour in this figure legend, the reader is referred to the web version of this article.)

**Fig. 20:**
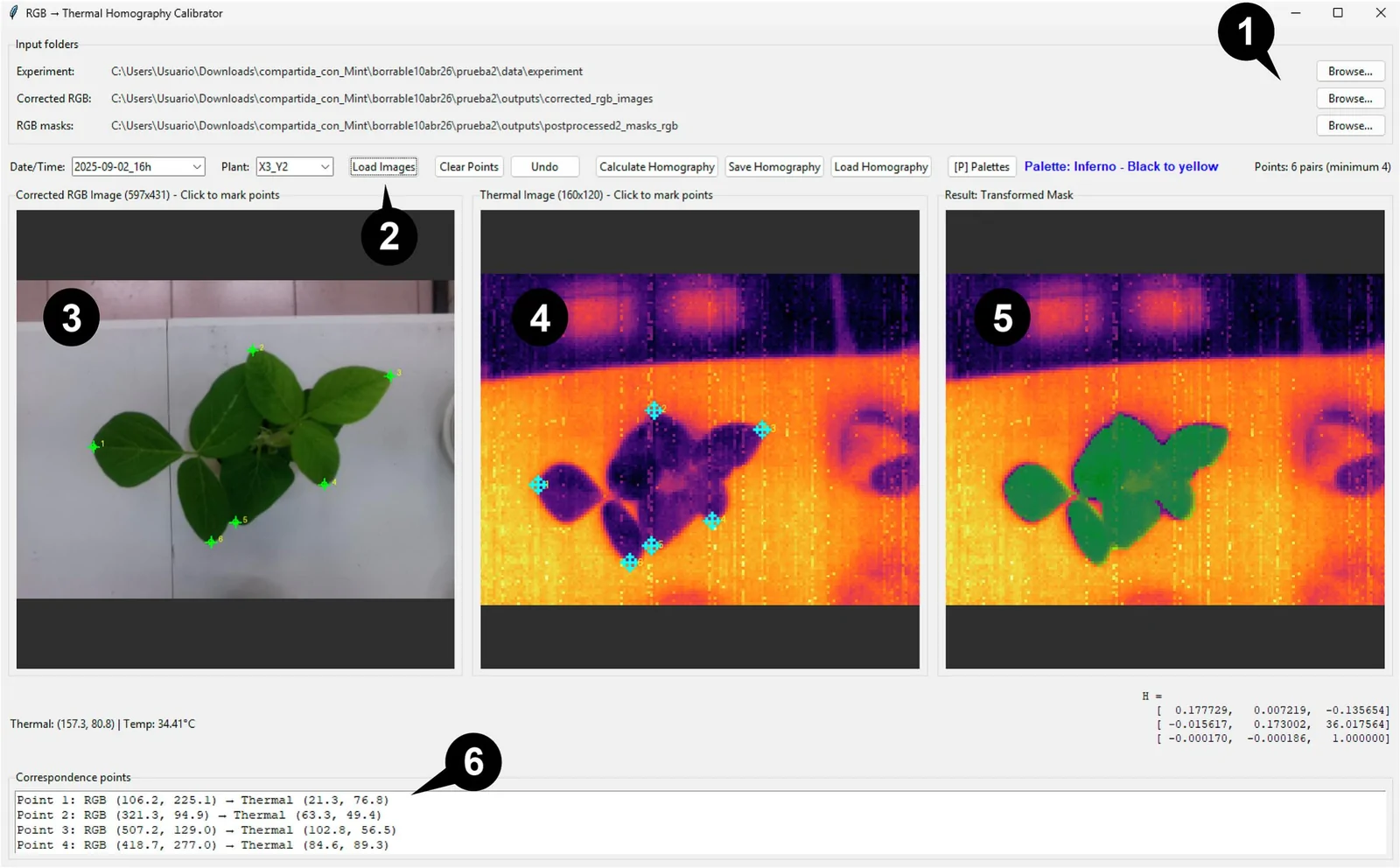
Tool for projecting the RGB image mask onto the thermographic image via homography (1_homography_calibrator.py). 1, menu for configuring directories containing the thermographic images, the geometrically corrected RGB and the processed RGB masks. 2, toolbar for loading images, deleting reference points, and calculating and loading homography computations. 3, RGB image with user-defined reference points. 4, thermographic image with corresponding reference points. 5, homography-projected RGB mask overlaid on the thermal image. 6, correspondence point pairs and computed homography matrix.

**Fig. 21:**
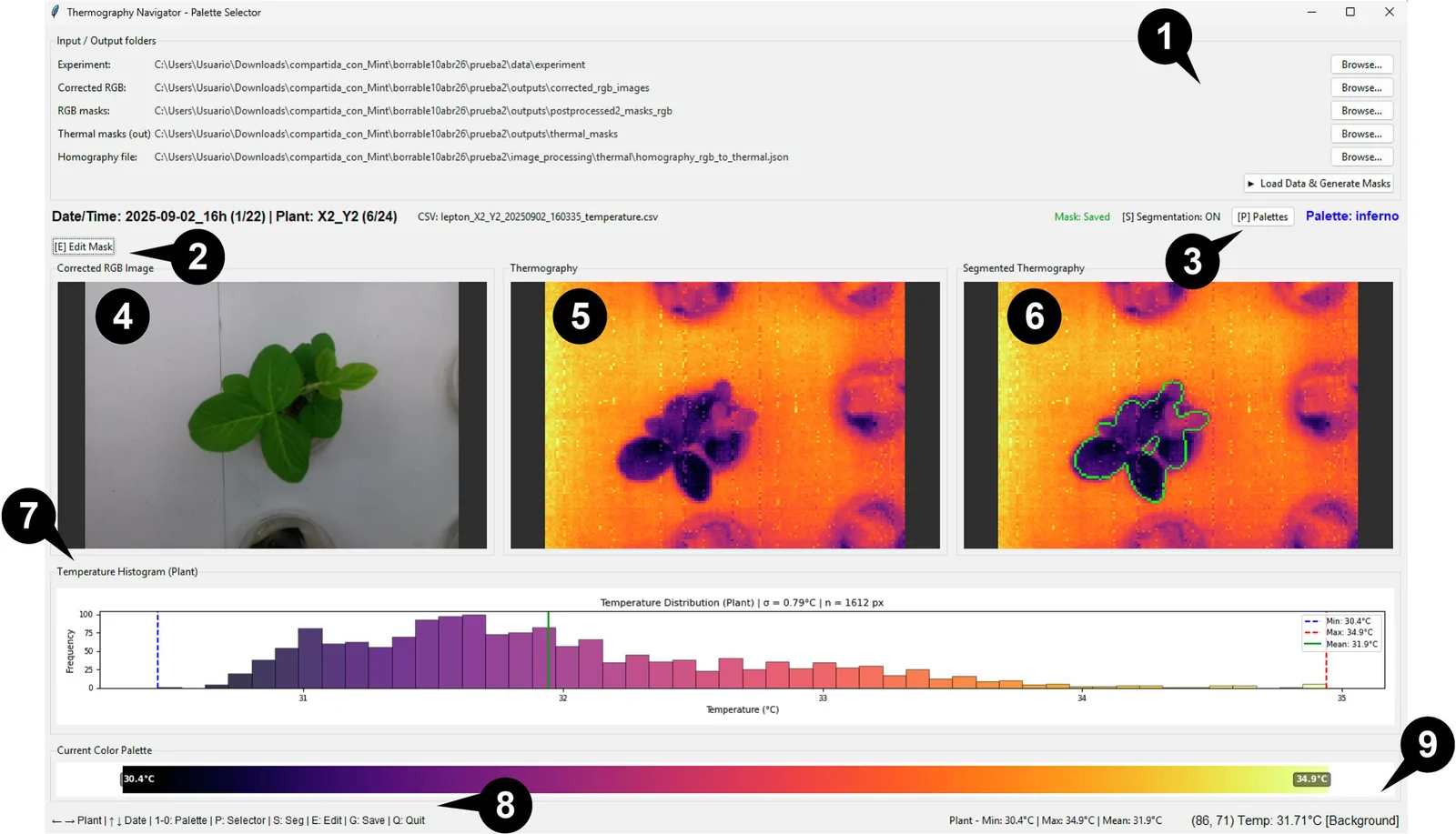
Graphical interface of the software developed for exploring thermographic images (2_palette_navigator.py). This software is used to perform the homographic conversion of the mask generated from the RGB image onto the lower-resolution thermographic images. This graphical interface allows inspection of the accuracy of this conversion, thereby identifying which thermographs require manual mask modification. At the bottom, a histogram of the temperature values acquired by the pixels encompassed by the mask in the thermograph is displayed. 1, panel for configuring input/output directory paths (experiment data, corrected RGB images, RGB masks, thermal mask output, and homography file) and loading data. 2, button to activate the thermographic mask editing mode. 3, button to change the color palette used to display the thermography. 4, RGB image. 5, thermography. 6, mask overlaid on the thermographic image. 7, temperature histogram of the points contained within the thermographic mask. 8, summary of the most commonly used commands. 9, thermal values of the thermographic mask and of the pixel under the cursor on the thermography.

**Fig. 22:**
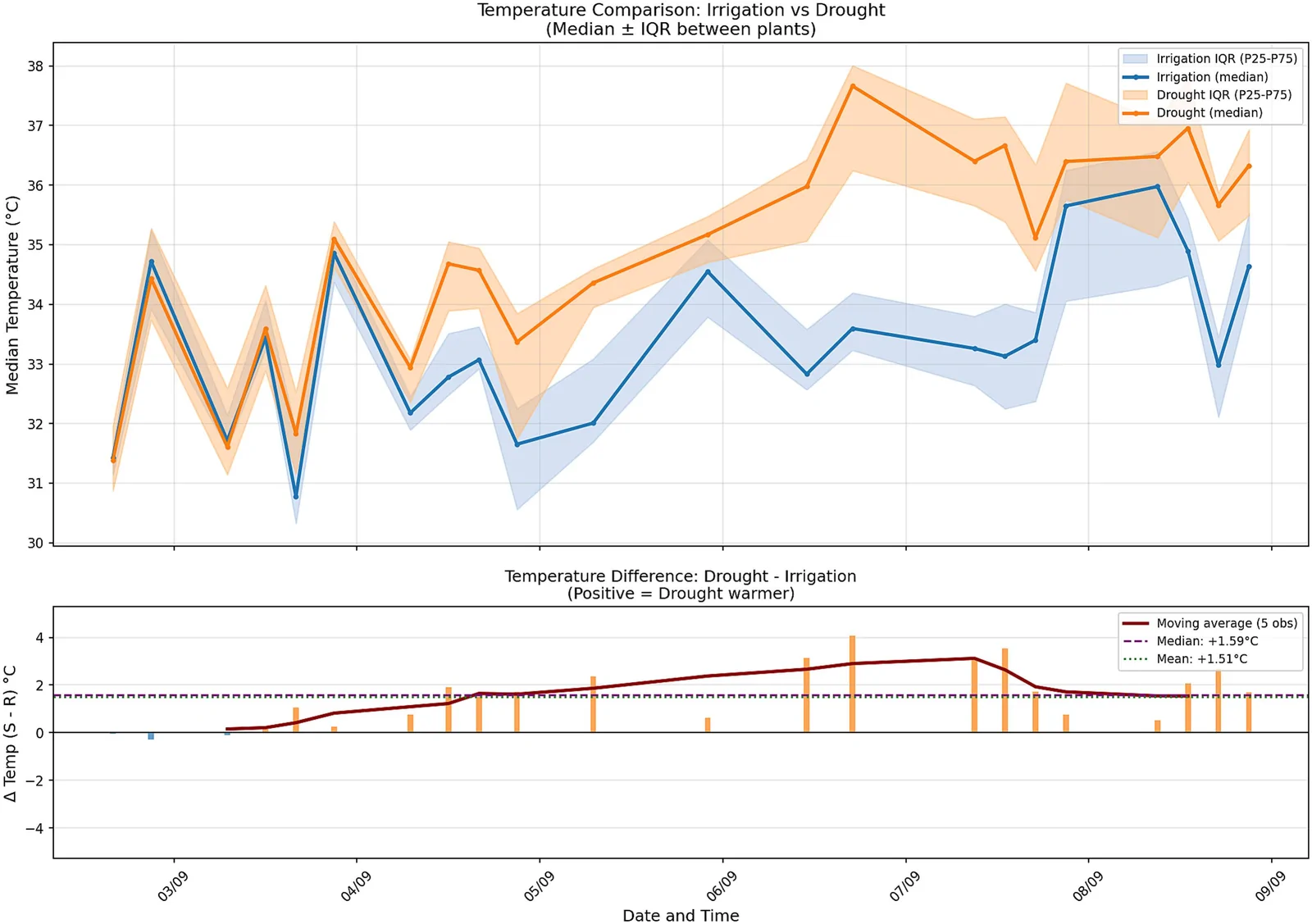
Canopy temperature comparison between irrigated (blue) and drought-stressed (orange) treatments during the validation period. Upper panel: Median temperature per acquisition session, with bands indicating the interquartile range (IQR, P25-P75) among the 12 plants per treatment. Lower panel: Temperature difference between treatments (Drought − Irrigated, labelled S − R on the axis) per session. Orange bars indicate sessions where drought-stressed plants exhibited higher temperatures; blue bars indicate the opposite. The red line represents the rolling mean (5-observation window). (For interpretation of the references to colour in this figure legend, the reader is referred to the web version of this article.)

### 7.1 Leaf area dynamics from RGB imaging

The image processing pipeline (Fig. 16) comprises three main stages: (1) geometric correction of RGB images to remove lens distortion, (2) semantic segmentation using deep learning to generate plant masks, and (3) quantitative analysis of leaf area and canopy temperature. RGB images were corrected for barrel distortion using intrinsic camera parameters obtained via OpenCV’s chessboard calibration method [32]. A U-Net architecture [33] with ResNet34 encoder was trained on 100 images manually labeled using the Supervisely platform (training pipeline: 2_plant_segmentation_training.ipynb), which was then used to segment 528 images across 24 plants and 22 timepoints. For thermal analysis, a projective transformation (homography) was calculated to map RGB segmentation masks onto the thermal camera resolution, providing an initial approximation of plant pixel locations in the thermographic images. Due to differences in resolution and viewing geometry between sensors, the projected masks required manual curation in most cases using a dedicated editing tool (2_palette_navigator.py). The homography-based projection thus served primarily as a starting reference to accelerate the manual delineation of plant regions in the thermal images, from which temperature statistics were subsequently extracted. In its current implementation, the platform is best suited for small- to medium-sized plants in controlled environments, where plant height and working distance remain compatible with the selected optical configuration. Likewise, the use of a FLIR Lepton 3.5 thermal sensor (160 × 120 px) provides sufficient resolution for canopy-level thermal assessment, although manual curation of projected masks may still be required in applications demanding precise boundary definition. These aspects should be understood as design trade-offs associated with the system’s emphasis on affordability, modularity, and ease of replication.

Acquired images were segmented using a U-Net model trained on 100 semi-automatically annotated images (80 training, 20 validation) using the Smart Tool of the Supervisely web platform, with manual refinement where needed. To improve generalization from this limited training set, online data augmentation was applied exclusively to the training partition using the Albumentations library, including random horizontal and vertical flips, 90° rotations, affine transformations (shift, scale, rotation), brightness and contrast adjustments, and Gaussian noise. Segmentation masks were post-processed using the Jupyter notebook script 3_mask_postprocessing_adaptive_bbox.ipynb to isolate the central plant in each image, applying adaptive bounding box filtering and morphological watershed separation to remove neighboring plants from the field of view. A small number of masks where automated post-processing failed to fully resolve plant overlap were manually corrected using the script 5_image_mask_viewer_napari.py which includes the tool Napari. The complete segmentation and post-processing pipeline is available at the repository associated with this paper.

The segmentation model was trained for 50 epochs. Training and validation loss curves showed consistent convergence, both decreasing from initial values above 0.7 to approximately 0.05, with no divergence between them, indicating the absence of overfitting (Fig. 17a). The Intersection over Union (IoU) metric for the plant class reached 0.95 on the validation set by epoch 30 and remained stable thereafter (Fig. 17b). Similarly, the Dice coefficient on the validation set plateaued at 0.975 after approximately 25 epochs (Fig. 17c). The close agreement between training and validation metrics throughout the training process, together with the final validation IoU of 0.95 and Dice score of 0.975, indicate that the model achieved robust and accurate segmentation of plant tissue. From a hardware validation perspective, these results confirm that the images produced by the platform are of sufficient quality to support automated deep learning-based analysis pipelines with high segmentation accuracy, a key requirement for reliable high-frequency phenotyping.

To demonstrate the platform’s capability for automated phenotyping, a water deficit experiment was conducted with 24 soybean plants, 20 days after sowing, arranged in a 6 × 4 grid, with 12 plants under regular irrigation and 12 under drought stress. Irrigation was withheld starting 20 days after sowing (day 0 of the experiment). RGB images were captured at regular intervals over 7 days and segmented using a U-Net model to extract binary masks of each plant (Fig. 18). The projected leaf area was then quantified from the segmented masks for each individual plant across the time series (Fig. 19). The resulting growth curves clearly discriminated between irrigated and drought-stressed plants, with the latter showing a marked decline in projected leaf area from approximately day 5 (September 7) of the experiment, demonstrating the platform’s ability to capture physiologically relevant phenotypic responses in a non-invasive and automated manner. The platform made it possible to detect the physiological phenomenon of nyctinasty, a rhythmic movement of soybean leaves regulated by the day–night cycle, in which leaflets are positioned more horizontally during the day to maximize light interception, while at dusk they fold and adopt a more vertical or downward orientation. This can be clearly observed as a reduction in leaf area during the night. In the non-irrigated plants, this phenomenon is observed to a lesser extent from the fifth day (September 7) onward, which is consistent with previous evidence indicating that water stress attenuates daily leaf movements, including nyctinasty. This type of movement is driven by cellular turgor, which is directly linked to the plant’s water status and is affected under conditions of water stress [34].

On the fourth day (September 6) of the experiment, a power outage occurred, causing the plants to remain without light for a longer period until illumination was restored; as a consequence, leaf area reached a minimum at that time. Notably, this event highlights the system’s sensitivity, as it was able to detect an anomaly in the growth conditions, specifically the unexpected interruption of illumination, demonstrating its potential for monitoring and identifying environmental disturbances during experiments.

### 7.2 Canopy temperature dynamics from thermal imaging

The FLIR Lepton thermal sensor module generates six output files per acquisition. Three of these files, a NumPy array (.npy), a TIFF image (.tiff), and a raw Y16 binary file (.y16), contain identical radiometric data stored as unsigned 16-bit integers in centikelvin units. A pre-computed temperature file (.csv) provides the same data already converted to degrees Celsius (°C = raw value / 100 − 273.15), with each cell corresponding to a single pixel in the 160 × 120 thermal matrix. The remaining two files are 8-bit visualizations: a pseudocolor image (.jpg) rendered with a colormap for qualitative inspection, and a grayscale image (.jpg) for quick visual reference. Because JPEG compression is lossy and the 8-bit encoding maps the full temperature range to only 256 discrete levels, these visualization files are unsuitable for quantitative analysis. For all subsequent thermal analyses (including plant segmentation, temperature extraction, and statistical characterization) we used the CSV temperature files, which preserve the full radiometric resolution of the sensor in physical units (°C) and can be directly loaded as numerical arrays without requiring any additional conversion step. These values were used as radiometric temperature estimates suitable for relative treatment comparisons under the controlled and constant imaging geometry of the present experimental setup.

The integration between RGB-based segmentation and thermographic analysis is implemented through a dedicated software pipeline, as illustrated in Fig. 20, Fig. 21, Fig. 22. Fig. 20 shows the graphical interface of the homography calibration tool (1_homography_calibrator.py), which enables the projection of RGB-derived plant masks onto thermographic images by defining corresponding reference points between both modalities. This projection provides an approximate spatial alignment between sensors, serving as a starting reference for the subsequent manual curation of thermal masks using the exploration tool described below.

Fig. 21 presents the thermographic exploration interface (2_palette_navigator.py), which allows users to visualize the homography-projected RGB masks overlaid on thermal images, manually edit the mask boundaries to correct misalignments, adjust visualization parameters, and inspect temperature distributions through pixel-level histograms extracted from masked regions. This tool was used to curate the thermal masks for each plant and timepoint, ensuring that temperature statistics were computed predominantly from pixels corresponding to plant tissue.

Finally, Fig. 22 illustrates the application of this workflow to thermographic datasets, demonstrating how the combined use of homography-based mask projection and manual curation enables the extraction of temperature data predominantly associated with plant tissues. This modular approach facilitates reproducible integration of RGB and thermal data and can be adapted to other multimodal imaging systems.

In summary, the platform presented here demonstrates that low-cost, open-source hardware can be combined with advanced image analysis to generate reproducible, time-resolved phenotypic data under controlled conditions. Beyond its immediate application to leaf area dynamics and canopy temperature, the system provides a flexible framework for integrating additional sensors, analytical pipelines, and experimental designs. For instance, RGB-based greenness indices such as the Green Index [25] could be readily computed from the images already acquired by the platform, providing a non-destructive proxy for chlorophyll trends alongside the existing leaf area and thermal analyses. Its main contribution is not to replicate high-throughput commercial phenotyping facilities, but to provide an accessible and reproducible solution for multimodal, high-frequency phenotyping in growth chambers. Future developments may focus on integrating automated irrigation and gravimetric monitoring modules, expanding three-dimensional phenotyping capabilities, and linking acquired data with predictive models, thereby contributing to a more comprehensive and scalable understanding of plant responses to environmental stress. This adaptability positions the platform as a useful bridge between manual controlled-environment measurements and more complex physiological studies.

## Declaration of generative AI and AI-assisted technologies in the manuscript preparation process

During the preparation of this work, the authors used Claude (Anthropic), ChatGPT (OpenAI), and Gemini (Google) to assist with software-code development and debugging and refinement of the manuscript text. After using these tools, the authors reviewed and edited the content as needed and take full responsibility for the content of the published article.

## CRediT authorship contribution statement

**Marcel Bentancor:** Writing – review & editing, Writing – original draft, Visualization, Validation, Software, Methodology, Investigation, Funding acquisition, Conceptualization. **Esteban Casaretto:** Writing – review & editing, Writing – original draft, Visualization, Validation, Methodology, Investigation, Funding acquisition, Conceptualization. **Omar Borsani:** Supervision, Resources, Funding acquisition, Conceptualization. **Gastón Quero:** Visualization, Funding acquisition. **Mercedes Muñoz:** Software.

## Declaration of competing interest

The authors declare that they have no known competing financial interests or personal relationships that could have appeared to influence the work reported in this paper.
